# Intermittent Supplementation With Fisetin Improves Physical Function and Decreases Cellular Senescence in Skeletal Muscle With Aging: A Comparison to Genetic Clearance of Senescent Cells and Synthetic Senolytic Approaches

**DOI:** 10.1111/acel.70114

**Published:** 2025-05-28

**Authors:** Kevin O. Murray, Sophia A. Mahoney, Katelyn R. Ludwig, Jill H. Miyamoto‐Ditmon, Nicholas S. VanDongen, Nirad Banskota, Allison B. Herman, Douglas R. Seals, Robert T. Mankowski, Matthew J. Rossman, Zachary S. Clayton

**Affiliations:** ^1^ Department of Integrative Physiology University of Colorado Boulder Boulder Colorado USA; ^2^ Intramural Research Program National Institute on Aging, National Institutes of Health Baltimore Maryland USA; ^3^ Division of Gerontology, Geriatrics and Palliative Care, Department of Medicine University of Alabama at Birmingham Birmingham Alabama USA; ^4^ Division of Geriatrics University of Colorado Anschutz Medical Campus Aurora Colorado USA

**Keywords:** flavonoid, motor function, natural senolytic, senescence associated secretory phenotype, skeletal muscle senescence, transcriptome

## Abstract

Excess cellular senescence contributes to age‐related increases in frailty and reductions in skeletal muscle strength. In the present study, we determined the efficacy of oral intermittent treatment (1 week on—2 weeks off—1 week on) with the natural flavonoid senolytic fisetin to improve frailty and grip strength in old mice. Further, the effects of fisetin on physical function were evaluated in young mice. We performed bulk RNA sequencing of quadricep skeletal muscle to determine the cell senescence‐related signaling pathways modulated by fisetin. We also assessed the relative effects of fisetin on frailty and grip strength with aging in comparison with two other well‐established approaches for the removal of senescent cells: (1) genetic‐based clearance of excess senescent cells in old p16‐3MR mice, a model that allows for clearance of p16‐positive (p16+) senescent cells, and (2) oral intermittent treatment with the synthetic pharmacological senolytic ABT‐263 in old mice. We found that fisetin mitigated the adverse changes in frailty and grip strength with aging. Fisetin had no effects in young mice. The improvements in frailty and grip strength in old mice were accompanied by favorable modulation of the skeletal muscle transcriptome, including lower abundance of cellular senescence‐related genes (e.g., *Cdkn1a* and *Ddit4*). Improvements in frailty and grip strength with fisetin were comparable to those observed with genetic‐based clearance of excess p16+ senescent cells and treatment with ABT‐263. Taken together, our findings provide proof‐of‐concept support for fisetin as a senolytic strategy to improve physical function with aging.

## Graphical abstract

Supplementation with the natural senolytic agent fisetin attenuated age‐related declines in physical function in mice. Better physical function with fisetin was accompanied by lower expression of cell senescence‐related genes. Fisetin treatment had comparable effects on physical function as genetic clearance of senescent cells and synthetic senolytic therapy.

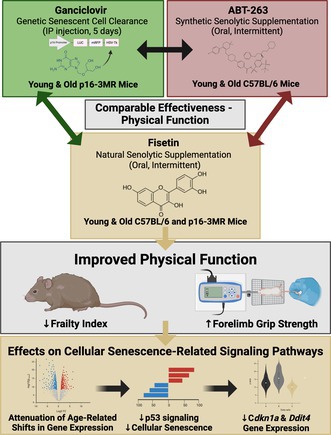

## 1 Introduction

Advancing age is associated with reductions in physical function (Grevendonk et al. 2021; Manning et al. 2024), characterized in part by lower skeletal muscle mass and strength (Bohannon 2019; Celis‐Morales et al. 2018), that contribute to the development of frailty (Cesari et al. 2006). Frailty, in turn, increases the risk of functional limitations, disabilities, loss of independence, and all‐cause mortality with aging (Court et al. 2025). As such, identifying effective interventions that promote healthy aging by reversing age‐related declines in physical function to reduce the risk of frailty and future disability is a biomedical research priority.

Cellular senescence refers to a state of largely permanent cell cycle arrest that occurs in response to multifaceted stress stimuli (Campisi and di d'Adda Fagagna 2007; Kuehnemann et al. 2023). Under physiological conditions, cellular senescence is critical for homeostatic processes such as wound healing (Demaria et al. 2014) and cancer suppression (Campisi 2013; Campisi and di d'Adda Fagagna 2007) in part through the senescence‐associated secretory phenotype (SASP), which includes a milieu of cytokines, chemokines, metabolites, and growth factors secreted by senescent cells (Campisi 2013; Campisi and di d'Adda Fagagna 2007; Demaria et al. 2014). However, senescent cells accumulate in excess with aging and promote physiological dysfunction, including declines in skeletal muscle strength (Baker et al. 2016; Dungan et al. 2022a, 2022b; Englund et al. 2023; Xu et al. 2018; Zhang et al. 2022) and increases in frailty (Wang et al. 2024). Indeed, genetic‐ and pharmacological‐based clearance of excess senescent cells can improve skeletal muscle strength and reduce frailty in old mice (Dungan et al. 2022a, 2022b; Xu et al. 2018; Zhang et al. 2022), suggesting that decreasing excess senescent cell burden may be a promising therapeutic approach to reduce frailty and increase skeletal muscle strength with aging.

Although select pharmacological approaches targeting senescent cells (i.e., senolytics) have advanced to clinical trials, safe and effective senolytic strategies that improve physical function have not been established in humans (Hickson et al. 2019; Justice et al. 2019) and there remains a need to identify new senolytic approaches with translational potential to reverse age‐associated physical dysfunction. Fisetin is a flavonoid compound with senolytic properties that is found in commonly consumed foods (Bondonno et al. 2019; Khan et al. 2013) and thus has a high likelihood for translation in the context of healthy aging. Although fisetin is not present in foods in high enough concentrations to exert senolytic effects, administration of fisetin at high doses over short periods can improve physiological function (Farr et al. 2024). Indeed, we have shown that late‐life oral intermittent fisetin supplementation—to clear excess senescent cells without disrupting the basal physiological processes supported by cellular senescence and to reflect the supplementation paradigm utilized in clinical trials (Farr et al. 2024; Justice et al. 2019)—reduces vascular cell senescence and improves vascular function in old mice (Mahoney et al. 2024), whereas others have shown fisetin treatment extends health span and lifespan in mice without any apparent adverse effects (Tavenier et al. 2024; Yousefzadeh et al. 2018). However, it is unknown if fisetin treatment improves skeletal muscle strength and frailty with advancing age. Further, the cellular‐molecular signaling pathways targeted by fisetin in skeletal muscle that might underlie the senolytic effects of fisetin are unknown.

Here, we sought to obtain preclinical evidence of the efficacy of fisetin by employing a mouse model of age‐related physical dysfunction and assessing the ability of oral intermittent treatment with fisetin to mitigate age‐associated increases in frailty and declines in grip strength in old male and female mice. We also determined the impact of fisetin treatment on age‐associated changes in the skeletal muscle transcriptome, focusing on the effects of fisetin on cell senescence‐related signaling pathways in old mice to determine the molecular mechanisms by which fisetin may affect skeletal muscle. Finally, we compared the effects of treatment with fisetin on frailty and grip strength in old mice with the effects of genetic‐ and synthetic pharmacological‐based senescent cell clearance to gain insight into how the functional effects of fisetin as a natural compound might compare to more targeted senolytic approaches.

## 2 Methods

### 2.1 Ethical Approval and Animal Studies

All mice were housed in a conventional facility on a 12‐h light/dark cycle, given *ad libitum* access to an irradiated, fixed, and open standard rodent chow (Inotiv/Envigo 7917) and drinking water (Boulder, CO municipal tap water that underwent reverse osmosis and chlorination). Euthanasia via cardiac exsanguination was performed while maintained under anesthesia (inhaled isoflurane) following in vivo testing 3 to 4 weeks after the completion of the intervention periods. After cardiac exsanguination, the quadriceps muscles were excised, flash frozen in liquid nitrogen, and stored at −80°C.

Investigators were blinded to the treatment group for data collection and biochemical analyses. All animal protocols were approved by the University of Colorado (CU) Boulder Institutional Care and Use Committee (protocol no. 2618) and complied with the National Institutes of Health Guide for the Care and Use of Laboratory Animals (National Research Council (US) Committee for the Update of the Guide for the Care and Use of Laboratory Animals, 2011). Any deceased animals were found in the morning during daily health checks by our animal care staff. All mice were autopsied and there was no evidence of esophageal perfusion or fluid in the lungs. Further, there was no evidence of intervention‐induced toxicity as assessed during tissue dissection. The number of animals found deceased in each treatment group is reported in the methods section of the corresponding intervention (i.e., fisetin, p16‐3MR, ABT‐263).

### 2.2 Fisetin Study

For the intervention period, young (6–8 months) and old male (27 months) C57BL/6N wildtype and young (6–8 months) and old male (27 months) and female (29 months) p16‐3MR mice (on C57BL/6 background) were randomly assigned to receive vehicle (Veh; 10% EtOH, 30% PEG400 and 60% Phosal 50 PG) or fisetin (50 mg/kg/day in Veh). Differences in age between old male and female mice were to account for differences in median lifespan between sexes observed on C57BL/6 background (Flurkey et al. 2007). For wildtype mice, 18 mice received Veh and 19 mice received fisetin, and for p16‐3MR mice, 27 mice received Veh and 36 mice received fisetin. Treatment was administered via daily oral gavage using an intermittent dosing paradigm of 1 week on—2 weeks off—1 week on (Mahoney et al. 2024). Treatment groups were matched for baseline body weight.

Throughout this intervention period, ten old mice died (1 wildtype mouse and 9 p16‐3MR mice) as a result of typical age‐related attrition, which resulted in a final sample size of Young Veh, *N* = 9 (5 males [M]/4 females [F]); Young Fisetin, *N* = 11 (6 M/5F); Old Veh, *N* = 31 (23 M/8F); and Old Fisetin, *N* = 39 (31 M/8F).

### 2.3 p16‐3MR Study

p16‐3MR mice are animals that carry a trimodal fusion protein (3MR) under the control of the p16^INK4A^ promoter which allows for selective genetic clearance of excess p16+ senescent cells, back to young/basal levels, by administering the prodrug ganciclovir (GCV), as previously described (Clayton et al. 2023; Demaria et al. 2014, 2017; Mahoney et al. 2024).

Young (6–8 months) and old male (27 months) and female (29 months) p16‐3MR mice were studied. Mice were randomly assigned to receive Veh (sterile saline; young: *N* = 18, old: *N* = 21) or GCV (25 mg/kg in Veh; young: *N* = 17, old: *N* = 23) via intraperitoneal injection for five consecutive days, as described previously (Clayton et al. 2023; Demaria et al. 2014, 2017; Mahoney et al. 2024). Throughout this intervention period, six old mice died (Old Veh *N* = 3; GCV: *N* = 3) due to anticipated age‐associated attrition, which resulted in a final sample size of Young Veh, *N* = 18 (10 M/8F); Young GCV, *N* = 17 (10 M/7F); Old Veh, *N* = 18 (10 M/8F); and Old GCV, *N* = 20 (10 M/10F). Treatment groups were matched for baseline body weight.

### 2.4 ABT‐263 Study

Young and old male C57BL/6N mice were obtained from the National Institute on Aging colony (maintained by Charles River, Wilmington, MA) and studied at 6 and 27 months of age, respectively.

For the intervention period, mice were randomly assigned to receive Veh (10% EtOH, 30% PEG400 and 60% Phosal 50 PG; young: *N* = 18, old: *N* = 23) or ABT‐263 (50 mg/kg/day in Veh; young: *N* = 11, old: *N* = 18), a dose that we have previously shown to effectively reduce vascular senescent cell burden in old mice (Clayton et al. 2023). Treatment was administered via oral gavage using an intermittent dosing paradigm of 1 week on—2 weeks off—1 week on, as described previously (Clayton et al. 2023). Treatment groups were matched for baseline body weight.

Prior to or throughout the intervention period, 16 old mice died (prior to baseline testing: *N* = 8; vehicle dosing: *N* = 2; ABT‐263 dosing: *N* = 6) due to anticipated age‐related attrition. This resulted in a final sample size of Young Veh, *N* = 18; Young ABT‐263, *N* = 11; Old Veh, *N* = 15; and Old ABT‐263, *N* = 10.

### 2.5 Frailty

Frailty was assessed using a validated 31‐point index covering seven subdomains (Whitehead et al. 2014) that is similar and compares to clinical frailty indices in humans. Briefly, frailty was calculated and compared across age and treatment. A “0” represents the absence of a deficiency, “0.5” represents a mild deficit, and “1” represents a severe deficit. Of note, forelimb grip strength was one of the 31‐points within the frailty index and included within the physical/musculoskeletal subdomain.

### 2.6 Forelimb Grip Strength

Grip strength of the forelimbs was assessed using a customized grip strength device which contained a force transducer (0.5 kg, Imada PS Series, Northbrook IL, USA) attached to a trapeze grip of ~1.5 mm diameter, as previously described (Murray et al. 2024). Briefly, the mouse was grasped by the tail, suspended just above the trapeze bar, and lowered until it successfully grasped the bar with both forepaws. A gradual horizontal tug was then applied until the mouse released its grip. Five trials were taken with 30 s between trials. Trials in which the mouse forcefully jerked the bar rather than simply releasing its grasp were included.

### 2.7 RNA Sequencing

Messenger RNA (mRNA) was isolated from 30 mg of frozen quadriceps skeletal muscle from mice in each of the Young Veh, Old Veh, and Old Fisetin groups (*N* = 10/group; balanced for sex and mouse type [wildtype and p16‐3MR]) using Qiagen RNeasy Mini Kit (cat. Nos. 74,104 and 74,106) with TRIzol following mechanical homogenization with ceramic mortar and pestle on dry ice to keep the sample frozen. RNA concentration was measured using a NanoDrop spectrophotometer (Thermo Scientific 2000), and purity was determined by absorptions of 260/280, with all samples exhibiting ≥ 2.0. mRNA was then shipped to Novogene for bulk sequencing. At Novogene, mRNA was purified from total RNA using poly‐T‐oligo‐attached magnetic beads. After fragmentation, the first strand cDNA was synthesized using random hexamer primers, followed by the second strand cDNA synthesis. The library was checked using Qubit and real‐time PCR for quantification and a bioanalyzer for size distribution detection. Quantified libraries were pooled and sequenced on an Illumina NovaSeq 6000 S4. Our study team then performed differential expression using counted reads through the DESeq pipeline on the ExpressAnalyst platform (Ewald et al. 2024) and Kyoto Encyclopedia of Genes and Genomes (KEGG) pathway analyses. Visual representation of these data was constructed using GraphPad Prism v10.2.3. Principal component analysis was performed using ClustVis (Metsalu and Vilo 2015).

### 2.8 Deconvolution Analysis

A deconvolution analysis of the bulk RNA‐sequencing data for the Young Veh, Old Veh, and Old Fisetin groups was performed using the R package BayesPrism v. 2.2.2 to identify cell‐type specific changes in gene expression (Chu et al. 2022). Specifically, we deconvoluted our bulk dataset utilizing a previously published dataset (Walter et al. 2024), which identified 29 cell types in mouse skeletal muscle. Mitochondrial genes, as well as chromosomes X and Y (sex‐specific) genes, were excluded from this analysis. Additionally, genes expressed in fewer than five cells were excluded in filtering. The filtered dataset was then used to perform the deconvolution and estimate cell type proportions. Finally, we applied DESeq2 v. 1.46. (Love et al. 2014) to the deconvoluted datasets to identify genes that showed differential expression across cell types.

### 2.9 Reverse Transcription Polymerase Chain Reaction (RT‐PCR)

mRNA was extracted from frozen quadriceps skeletal muscle tissue (*N* = 10/group) as described in the RNA sequencing section from Young and Old Veh‐treated (5 M/5F), old p16‐3MR GCV‐treated (5 M/5F), and old ABT‐263‐treated (10 M/0F) mice. Complement DNA (cDNA) was synthesized using the iScript cDNA synthesis kit (Bio‐Rad Laboratories, Hercules, CA). Transcripts of key cellular senescence and SASP markers (primer sequences reported below) were analyzed using a StepOnePlus Real‐Time PCR System (Applied Biosystems, Waltham, MA) in 96‐well plates, and SYBR green reagents (Thermo Fisher Scientific, Waltham, MA) were used as a master mix, as previously described by our laboratory (Mahoney et al. 2024). SimpleSeq DNA sequencing (Quintara Biosciences, Cambridge, MA) was used to validate PCR products. PCR outputs were analyzed by the ΔΔCt method, normalized to *Gapdh*, and then visually depicted relative to the Young Veh‐treated group (Table 1).

**TABLE 1:** Primers for reverse transcription polymerase chain reaction (RT‐PCR).

Gene	Species	Forward primer	Reverse primer
*Cdkn2a*	Mouse	CCCAACGCCCCGAACT	GCAGAAGAGCTGCTACGTGAA
*Cdkn1a*	Mouse	TTGCCAGCAGAATAAAAGGTG	TTTGCTCCTGTGCGGAAC
*Pai1*	Mouse	TGGAAGGGCAACATGACCAG	TCAGGCATGCCCAACTTCTC
*Lmnb1*	Mouse	GAGCCCCAAGAGCATCCAAT	CTGAGAAGGCTCTGCACTGT
*Gapdh*	Mouse	AAGGTCATCCCAGAGCTGAA	CTGCTTCACCACCTTCTTGA

### 2.10 Statistical Analyses

Statistical analyses were performed with G*Power 3.1 and GraphPad Prism (v10; La Jolla, CA). Using the Grubbs test (*α* < 0.05), outliers were identified and removed. We observed select outliers in the RT‐PCR and RNA sequencing data but not the grip strength and frailty variables. Frailty, grip strength, and RT‐PCR expression were analyzed by ordinary one‐way ANOVAs with Tukey's multiple comparisons test. RNA sequencing data was analyzed using the DESeq pipeline on Express Analyst (Ewald et al. 2024) and reported as Log_2_[FoldChange (FC)] and unadjusted *p*‐values. Genes were considered differentially expressed between groups if they met both parameters of Log_2_FC > ± 0.5 and *p*‐value < 0.05. Pathways were considered significant with a false discovery rate (FDR)‐adjusted *p*‐value < 0.2. Data are presented as mean ± SEM. Statistical significance was set a priori at *⍺* < 0.05.

## 3 Results

### 3.1 Mouse Characteristics

Select morphological characteristics for all groups have been previously reported (Clayton et al. 2023; Mahoney et al. 2024). In addition, we assessed the mass of the soleus, gastrocnemius, tibialis anterior, and quadriceps muscles for the present study. Notably, age‐related differences in body mass were not observed (Clayton et al. 2023; Mahoney et al. 2024), but skeletal muscle mass was lower in old mice compared with young mice in each group (Table 2). There were no effects of any treatment on morphological characteristics in young or old mice (Table 2) (Clayton et al. 2023; Mahoney et al. 2024). There were no sex differences in any outcomes assessed so the data were combined for all analyses (Clayton et al. 2023; Mahoney et al. 2024); however, male and female mice are indicated by different symbols in Figures.

**TABLE 2:** Mouse Skeletal Muscle Masses. The mass of soleus, gastrocnemius, tibialis anterior, and quadriceps skeletal muscles was measured after each intervention period. Mice within young vehicle (YV) or young treatment (YT) (~6 months of age); mice within old vehicle (OV) or old treatment (OT) (~27 months of age). Male (M) and female (F) mice within each experimental cohort.

Group	Fisetin				GCV				ABT‐263			
Treatment	YV	YF	OV	OF	YV	YG	OV	OG	YV	YA	OV	OA
Number (M/F)	9 (5/4)	11 (6/5)	31 (23/8)	39 (31/8)	18 (10/8)	17 (10/7)	18 (10/8)	20 (10/10)	18 (18/0)	11 (11/0)	15 (15/0)	10 (10/0)
Soleus mass, mg	16.3 ± 0.6	16.4 ± 0.8	14.1 ± 1.0^a^	14.4 ± 0.8^a^	15.3 ± 0.8	15.2 ± 0.6	13.8 ± 0.8^a^	13.3 ± 0.9^a^	16.7 ± 1.6	17.8 ± 1.2	11.1 ± 0.7^a^	11.3 ± 0.9^a^
Gastrocnemius mass, mg	237.2 ± 6.2	230.8 ± 9.0	181.6 ± 12.6^a^	179.1 ± 11.0^a^	230.0 ± 13.9	220.1 ± 9.8	185.2 ± 12.5^a^	181.3 ± 12.9^a^	231.8 ± 17.6	231.6 ± 30.7	195.8 ± 15.9^a^	173.2 ± 11.9^a^
Tib. Anterior mass, mg	111.2 ± 4.1	109.3 ± 6.7	97.8 ± 4.6^a^	95.8 ± 3.2^a^	118.1 ± 11.7	102.6 ± 7.2	107.2 ± 6.0^a^	102.0 ± 5.1^a^	125.7 ± 10.5	123.8 ± 8.5	94.2 ± 9.7^a^	92.8 ± 11.4^a^
Quadriceps mass, mg	284.6 ± 10.1	282.9 ± 16.2	218.9 ± 9.7^a^	224.4 ± 10.0^a^	281.5 ± 10.0	278.1 ± 17.9	231.0 ± 9.9^a^	235.3 ± 11.4^a^	311.2 ± 9.6	333.4 ± 7.6	240.4 ± 16.0^a^	233.6 ± 11.6^a^

*Note:* Data are presented as mean ± SEM.

Abbreviation: Tib, tibialis.

^a^ *p* < 0.05 to: Young groups within respective experimental cohort.

### 3.2 Fisetin Improves Frailty Index Score and Grip Strength With Aging

**FIGURE 1:**
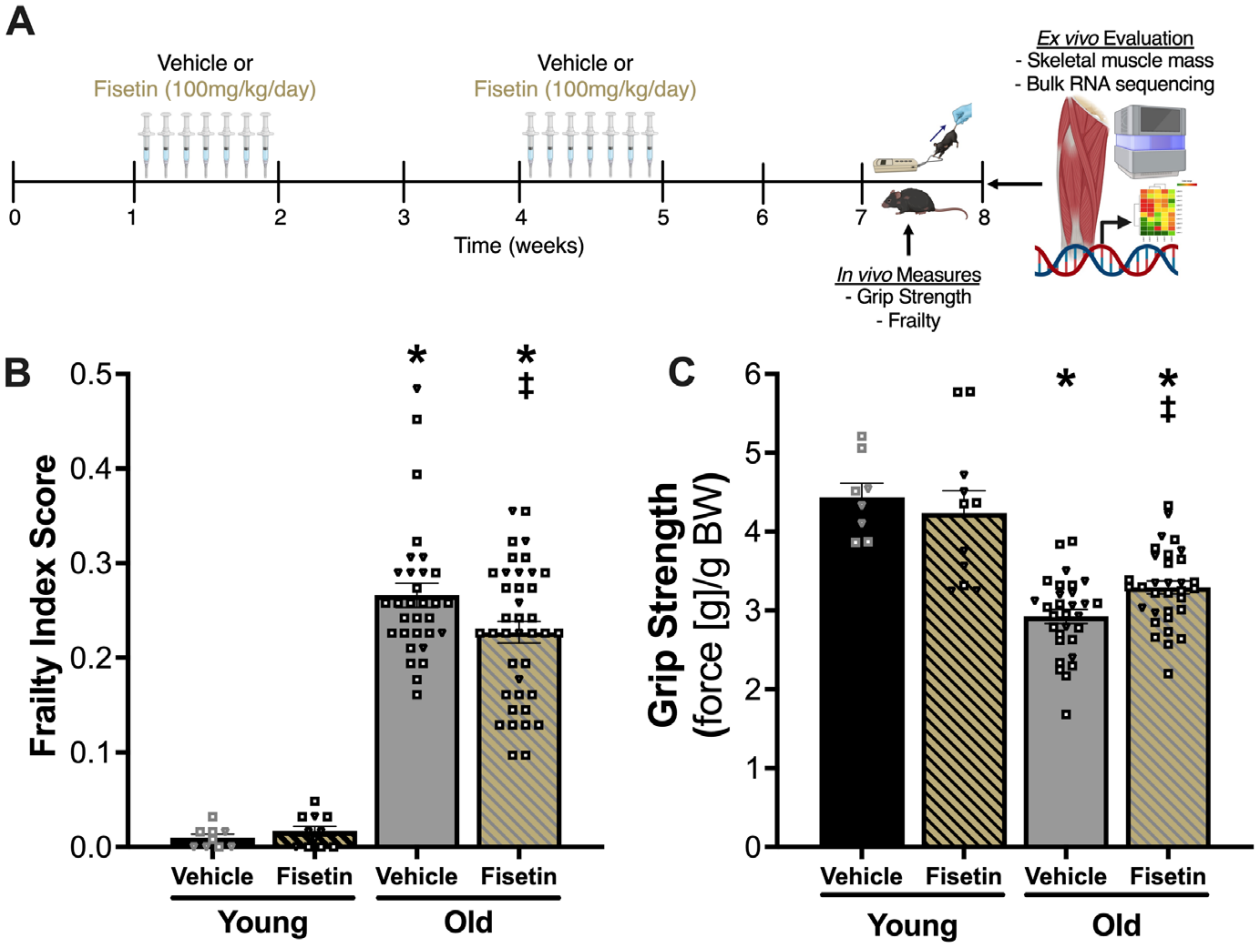
Oral treatment with fisetin attenuates differences in frailty index score and grip strength with aging in old mice. Experimental design (A). Frailty index score (B) and normalized grip strength (C). Squares = male mice, inverted triangles = female mice. Data presented as mean ± SEM. One‐way ANOVA corrected with Tukey's multiple comparisons test. *p* < 0.05: *both young groups; ^‡^old vehicle. Images made with Biorender.com.

To determine if intermittent supplementation with fisetin improves physical function with aging, we treated young and old mice with fisetin and quantified frailty and grip strength after the treatment period (Figure 1A). Old Veh‐treated mice (*N* = 30–31) had a higher frailty index score (0.27 ± 0.01 AU, *N* = 31) (Figure 1B; Table S1) and lower grip strength (2.90 ± 0.1 g/g BW, *N* = 30) (Figure 1C) compared with Young Veh‐treated mice (*0.01*± *0.01 AU*, *N* = 9; 4.40 ± 0.2 g/g BW, *N* = 8; both *p* < 0.0001). Old mice orally administered fisetin had 15% lower frailty index score (0.23 ± 0.01 AU, *N* = 39; *p* = 0.0264) (Figure 1B; Table S1) and 14% higher grip strength (3.30 ± 0.1 g/g BW, *N* = 30; *p* = 0.0038) (Figure 1C) compared with Old Veh‐treated mice. Fisetin attenuated the aging‐related changes in frailty index score (Figure 1B; Table S1) and grip strength (Figure 1C) by 18% and 40%, respectively.

Fisetin did not alter frailty index score (0.02 ± 0.01 AU, *N* = 11; *p* = 0.9949 vs. Young Veh) (Figure 1B; Table S1) or grip strength (4.2 ± 0.3 g/g BW, *N* = 11; *p* = 0.872 vs. Young Veh) (Figure 1C) in young mice. There was no influence of the mouse model (i.e., C57BL/6N or p16‐3MR) on responsiveness to the intervention, so data from each mouse model are combined.

### 3.3 Fisetin Treatment Attenuates Age‐Related Shifts in the Quadriceps Skeletal Muscle Transcriptome and Lowers Cellular Senescence in Old Mice

**FIGURE 2:**
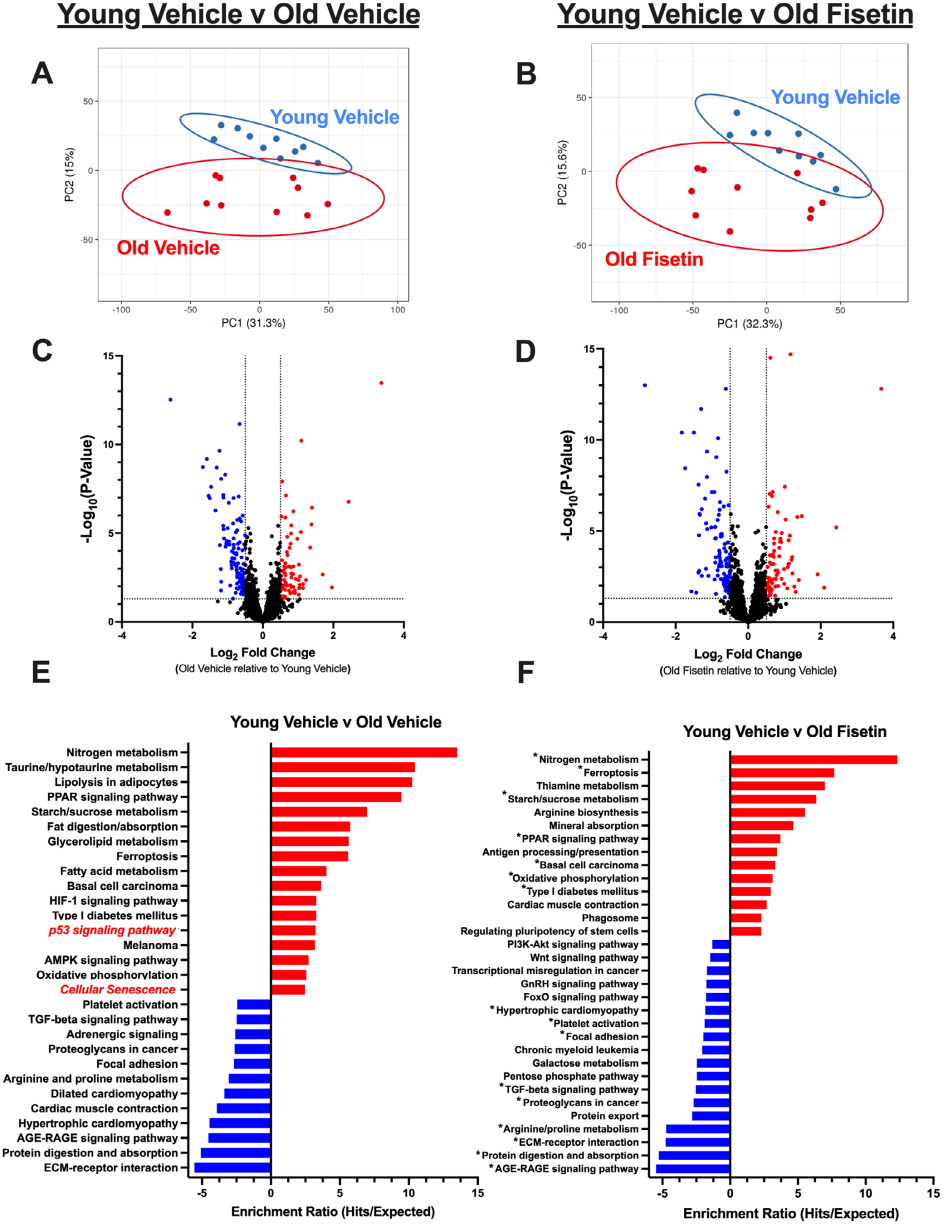
Global quadriceps skeletal muscle gene expression is favorably altered with fisetin treatment in old mice. Principal component analysis of skeletal muscle mRNA abundance with aging (A, B). Genes were determined differentially expressed (DEGs) using the parameters of Log2FoldChange > ± 0.5 and *p* < 0.05 (C, Old Vehicle vs. Young Vehicle; D, Old Fisetin vs. Young Vehicle). This was visualized via volcano plot. DEGs were analyzed using Kyoto Encyclopedia of Genes and Genomes (KEGG) to determine enriched molecular and physiological processes. Only significant KEGG pathways are shown (false discovery rate [FDR] < 0.2), * significant pathway in Young Vehicle vs. Old Vehicle comparison (E, Young Vehicle vs. Old Vehicle; F, Young Vehicle vs. Old Fisetin).

We next aimed to determine whether the attenuation of age‐related changes in frailty index score and grip strength following intermittent oral supplementation with fisetin are accompanied by favorable changes in the quadriceps skeletal muscle transcriptome, particularly in genes implicated in pathways associated with cellular senescence (*N* = 10/group, 5 M/5F). There were differences in global skeletal muscle gene expression between Young Veh‐ and Old Veh‐treated mice via principal component analysis (Figure 2A). A total of 185 differentially expressed genes (DEGs) were identified with aging in skeletal muscle (74 upregulated, red; 111 downregulated, blue) (Figure 2C). With fisetin treatment, the aging skeletal muscle phenotype was altered (191 genes; 83 upregulated, 108 downregulated) (Figure 2B,D) and there was less distinct separation from the young, as indicated by principal component analysis and less robust fold changes in DEGs.

To determine which molecular and physiological processes are implicated in aged skeletal muscle (Young Veh v Old Veh) and which molecular and physiological processes are affected by fisetin treatment in skeletal muscle of old mice (Young Veh v Old Fisetin), KEGG pathway analyses were performed on DEGs identified within each comparison. KEGG pathway analysis of DEGs between Young Veh and Old Veh indicated two enriched pathways related to senescent cell burden (in red) (Figure 2E). These pathways associated with cellular senescence were no longer enriched following fisetin treatment (Figure 2F).

**FIGURE 3:**
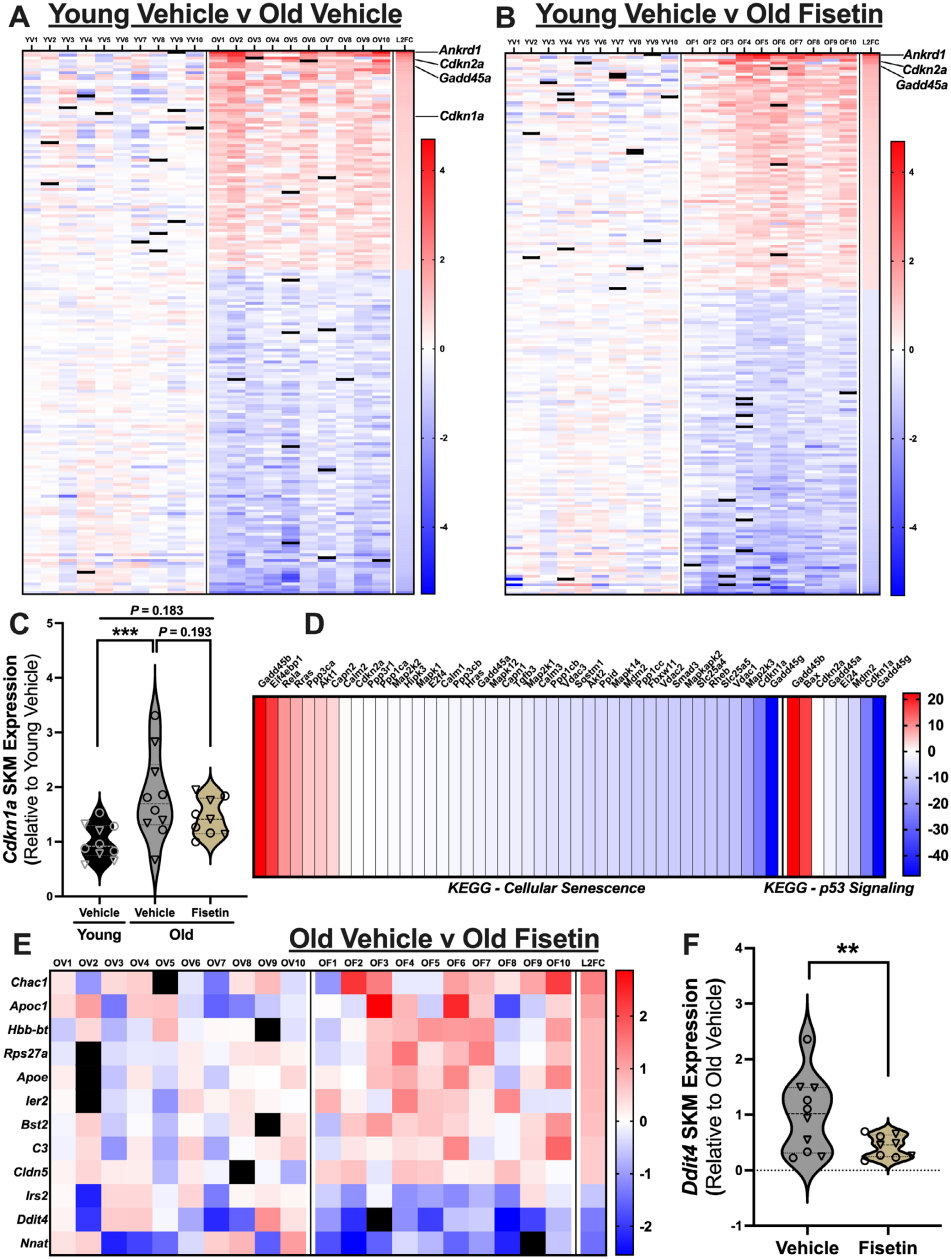
Fisetin treatment in old mice attenuates excess cellular senescent‐related gene expression in aged skeletal muscle. Individual values of differentially expressed genes (DEGs) relative to Young Vehicle (A, Old Vehicle vs. Young Vehicle; B, Old Fisetin vs. Young Vehicle); YV = Young Vehicle; OV = Old Vehicle; OF = Old Fisetin. Black boxes indicate the removal of an outlier (Grubbs test, *α* < 0.05) (A & B). Violin plot of transcript abundance of *Cdkn1a* (i.e., p21) in skeletal muscle analyzed by an ordinary one‐way ANOVA with Tukey's multiple comparisons test; ***p < 0.001; shown as Log_2_ fold change; squares = male mice, inverted triangles = female mice (C). Differences in the relative abundance of genes associated with cellular senescence and related pathways in old mice with and without fisetin treatment compared with Young Vehicle, e.g., gene “X” = [{(Old Fisetin/Young Vehicle)—(Old Vehicle/Young Vehicle)}*100] (D). Individual values of DEGs in old mice treated with fisetin (OF) relative to OV. Black boxes indicate the removal of an outlier (Grubbs test, *α* < 0.05) (E). Violin plot of transcript abundance of *Ddit4* (i.e., DNA damage inducible transcript 4 or *Redd1*); **p < 0.01; squares = male mice, inverted triangles = female mice (F).

Upon the observation that pathways related to cellular senescence were not enriched in the skeletal muscle of old mice relative to young mice following treatment with fisetin, we aimed to determine which DEGs associated with cellular senescence had been altered. We found that some of the most profound DEGs in skeletal muscle with aging are implicated in processes involved in cellular senescence (i.e., *Ankrd1*, *Gadd45a*, *Cdkn1a*, and *Cdkn2a*; Figure 3A & Data S1). Following treatment with fisetin, the marker of cell cycle arrest and senescent cell burden in skeletal muscle with aging (i.e., *Cdkn1a*; gene encoding p21, a cyclin‐dependent kinase inhibitor) was no longer a DEG (Figure 3B & Data S1). Further investigation of differences in *Cdkn1a* across groups revealed fisetin treatment ameliorated higher *Cdkn1a* gene expression in aged skeletal muscle by ~46%, and expression levels were similar to that observed in young mice (Figure 3C). Interestingly, we found that abundance levels of *Cdkn2a* (i.e., gene encoding p16, a cyclin‐dependent kinase inhibitor; Figure 3A,B, & Data S1), as well as *Ankrd1* and *Gadd45a* (Figure 3A,B, & Data S1), were not different with fisetin treatment.

To gain additional insight into how subtle differences in genes implicated in cellular senescence, p53 signaling, and/or the SASP may contribute to lower frailty and higher grip strength in skeletal muscle with aging following fisetin treatment, we quantified the relative transcript abundance (i.e., ratio) between Old Fisetin/Young Veh and Old Veh/Young Veh, respectively, of genes associated with senescence as identified by KEGG pathway analysis. We then assessed the absolute change in these ratios to determine the effect of fisetin treatment on markers of cell senescence in the skeletal muscle of old mice compared with young (Figure 3D). We found that 34 genes involved in these processes were less abundant (e.g., MAPK signaling [Anerillas et al. 2020] [7 total, e.g., *Map2k3, Mapk14*], oncogene signaling [Bianchi‐Smiraglia and Nikiforov 2012] [*Hras*], and growth arrest and DNA damage signaling [Zaidi and Liebermann 2022] [*Gadd45g*]) (Figure 3D). The few genes found more abundant also serve as integral components of skeletal muscle repair and regeneration (e.g., *Gadd45b* myoblast differentiation [Deng et al. 2021]); *Eif4ebp1* (metabolic protection, attenuates mitochondrial abnormalities, and reduces cellular senescence [Ang et al. 2022; Tsai et al. 2015]); *Rras* (inhibits pathological angiogenesis and maintains mature blood vessel function [Weber and Carroll 2021]); *Rela* (stimulates NFB pathway to induce p53‐mediated cell death/apoptosis [Khandelwal et al. 2011]); *Bax* (proapoptotic factor [Siu et al. 2005]) and *Capn2* (contributes to apoptosis and repairs damaged myofibrils/sarcolemma [Brulé et al. 2010]) and hypertrophy (e.g., *Akt1* mammalian‐target of rapamycin pathway and inhibits insulin resistance [Sasako et al. 2022]) (Figure 3D).

To more comprehensively understand how fisetin treatment alters cellular senescence‐related gene expression in skeletal muscle with aging, we then compared gene transcripts between old vehicle‐ vs. old fisetin‐treated mice. Twelve genes were different with fisetin treatment in old mice (Figures 3E, S2A, and S2C & Data S1). Of these 12 DEGs, 9 were upregulated and 3 downregulated. These genes are implicated in processes related to DNA damage (*Ddit4 or Redd1*; Figure 3F) (Coronel et al. 2022; Foltyn et al. 2019); skeletal muscle hypertrophy (*Bst2*, *Apoe*) (Agergaard et al. 2021; McKellar et al. 2021); insulin resistance and reactivity (*Apoc1*, *Irs2*) (Jong et al. 2001; Turaihi et al. 2018); and repair and regeneration of skeletal muscle and stem cell activation (*Rsp27a*, *Cldn5*, *C3*, *Chac1, Ier2*) (Li et al. 2023; Machado et al. 2021; Richards et al. 2022; C. Zhang et al. 2017). Further interrogation of these DEGs using KEGG analysis indicated that pathways involved in glutathione metabolism (upregulated) and microRNAs implicated in cancer (downregulated) (Figure S2B & Data S1) may also be modulated.

We then aimed to gain initial insight into the primary cell types affected by intermittent fisetin treatment in skeletal muscle. To do so, we conducted a deconvolution analysis (Chu et al. 2022). This analysis allows for the retrospective determination of which cell types are contributing to differences in gene expression in a bulk RNA sequencing data set (Chu et al. 2022). Based on the results from bulk RNA sequencing, we were most interested in elucidating, in a cell specific manner, the effects of fisetin treatment on reducing *Cdkn1a* expression with aging as well as the effects of fisetin treatment on lowering *Ddit4* expression in old mice. Of the cell types detected by the deconvolution analysis, we found that intermittent fisetin treatment did not alter the proportion of cells in skeletal muscle amongst comparison groups (Data S2). However, we found that treatment with fisetin may mitigate the age‐related increase in *Cdkn1a* expression (Data S2) and decrease in *Ddit4* expression (Data S2) in old mice in a similar manner across several different cell types, including myonuclei, pericytes, fibro‐adipogenic progenitors, and neural cells.

### 3.4 Studies to Determine the Comparative Effectiveness of Fisetin Treatment for Improving Physical Function in Old Mice

The relative efficacy of different senolytic strategies on physical function is not well understood. As such, we next sought to determine how the functional effects of fisetin compare to the effects of other well‐established approaches for removing excess senescent cells. To address this goal, we conducted two additional studies. The first study employed a genetic‐based clearance of excess senescent cells using the p16‐3MR mouse model (Clayton et al. 2023; Demaria et al. 2014) and the second study examined the effect of intermittent oral supplementation with the synthetic pharmacological senolytic agent ABT‐263 (Clayton et al. 2023). As in the fisetin study, we assessed frailty index score and grip strength after the intervention period for each study. We then compared the effects of these well‐established approaches (Clayton et al. 2023; Demaria et al. 2014) on frailty index score and grip strength with those observed following fisetin treatment. We also evaluated a small panel of well‐established senescent cell and SASP markers using RT‐PCR to determine the senolytic effect of each approach in skeletal muscle.

#### 3.4.1 Genetic‐Based Clearance of Excess p16+ Senescent Cells Improves Frailty Index Score, Grip Strength, and Skeletal Muscle Cellular Senescence in Old Mice

**FIGURE 4:**
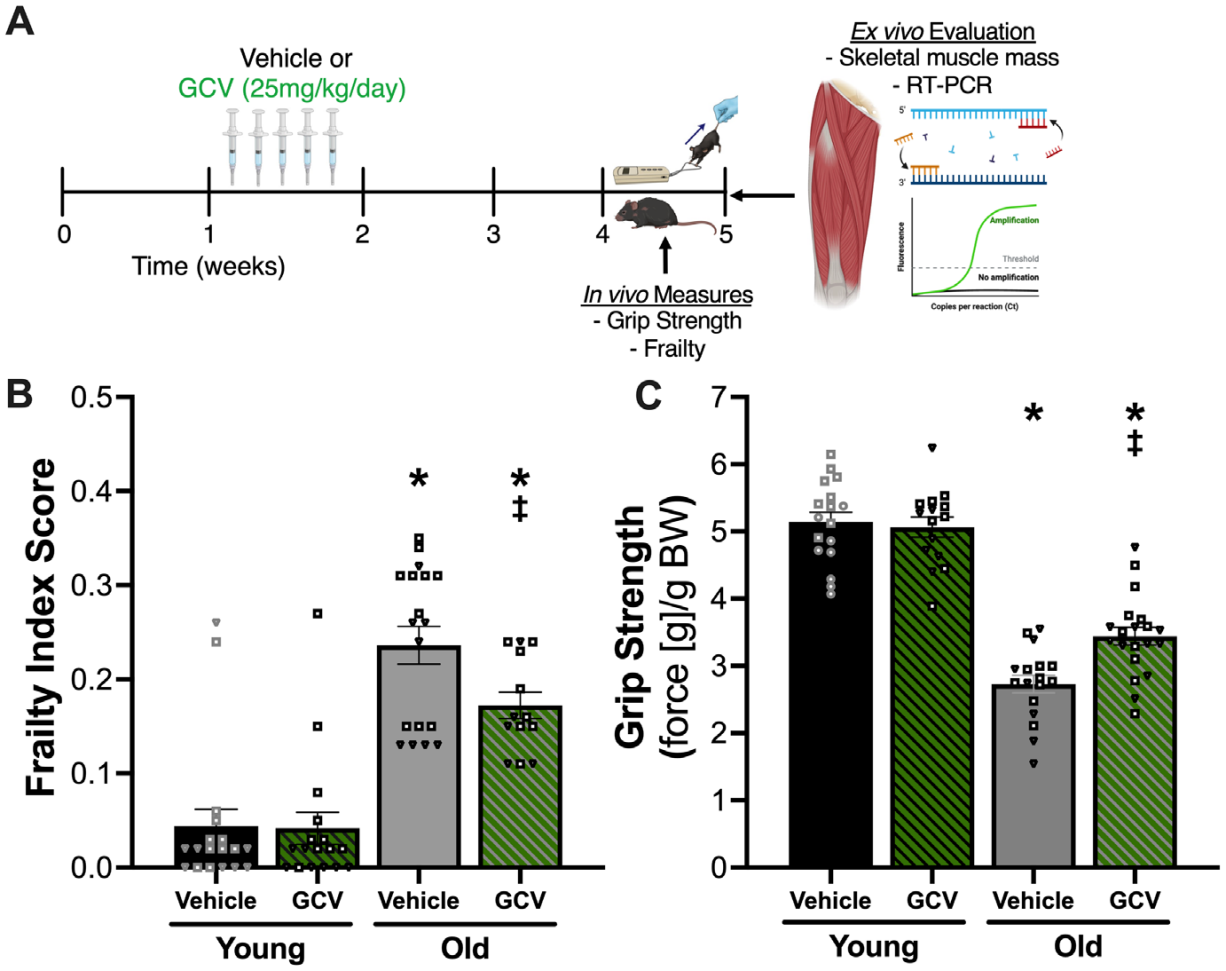
Genetic‐based clearance of excess p16+ senescent cells mitigates age‐related changes in frailty index score and grip strength in old mice. Experimental design (A). Frailty index score (B) and normalized grip strength (C). Squares = male mice, inverted triangles = female mice. Data presented as mean ± SEM. One‐way ANOVA corrected with Tukey's multiple comparisons test. *p* < 0.05: *both young groups; ^‡^old vehicle. Images made with Biorender.com.

Old p16‐3MR mice treated with the vehicle (Figure 4A) had a higher frailty index score (0.21 ± 0.02 AU, *N* = 18) (Figure 4B; Table S2) and lower grip strength (2.73 ± 0.13 g/g BW, *N* = 17) (Figure 4C) compared with Young Veh‐treated p16‐3MR mice (0.04 ± 0.02 AU, *N* = 18; 5.13 ± 0.15 g/g BW, *N* = 17; both *p* < 0.0001). Old p16‐3MR mice treated with the vehicle had higher expression of *Cdkn1a* (Figure S3A & Data S1) and *Cdkn2a* (Figure S3B & Data S1) compared with Young Veh‐treated p16‐3MR mice (*N* = 8‐10/group; *Cdkn1a*: *p* = 0.0001, *Cdkn2a*: *p* < 0.0001).

Old p16‐3MR mice treated with GCV (Figure 4A) (to activate the genetic clearance of excess p16+ senescent cells) had 27% lower frailty index score (0.16 ± 0.12 AU, *N* = 13; *p* = 0.0224) (Figure 4B; Table S2) and 22% higher grip strength (3.44 ± 0.13 g/g BW, *N* = 20; *p* = 0.0026) (Figure 4C) compared with old p16‐3MR mice treated with the vehicle. Genetic‐based clearance of excess p16+ senescent cells lessened the age‐associated differences in frailty index score (Figure 4B; Table S2) and grip strength (Figure 4C) by 25% and 33%, respectively. Additionally, old p16‐3MR mice treated with GCV (*N* = 10) had 44% lower expression of *Cdkn2a* (*p* = 0.0063; Figure S3B) compared with old p16‐3MR mice treated with the vehicle. Expression levels of *Cdkn2a* in the skeletal muscle of old GCV‐treated mice were similar to those observed in young mice (*p* = 0.1337; Figure S3B & Data S1). As anticipated with this mouse model, there was no effect of GCV treatment on *Cdkn1a* gene expression in the skeletal muscle of old mice (*p* = 0.7882; Figure S3A & Data S1).

Genetic‐based clearance of excess p16+ senescent cells did not influence frailty index score (0.04 ± 0.02 AU, *N* = 17; *p* = 0.9998 vs. Young Veh) (Figure 4B; Table S2) or grip strength (5.06 ± 0.15 g/g BW, *N* = 15; *p* = 0.9833 vs. Young Veh) (Figure 4C) in young mice. Further, there was no effect of age or treatment on *Pai1* (Plasminogen activator inhibitor‐1; Figure S3C & Data S1) or *Lmnb1* (Lamin B1; Figure S3D & Data S1) gene expression in skeletal muscle (*N* = 9‐10/group).

#### 3.4.2 Oral Synthetic Senolytic Therapy With ABT‐263 Enhances Frailty Index Score, Grip Strength, and Skeletal Muscle Cellular Senescence in Old Mice

**FIGURE 5:**
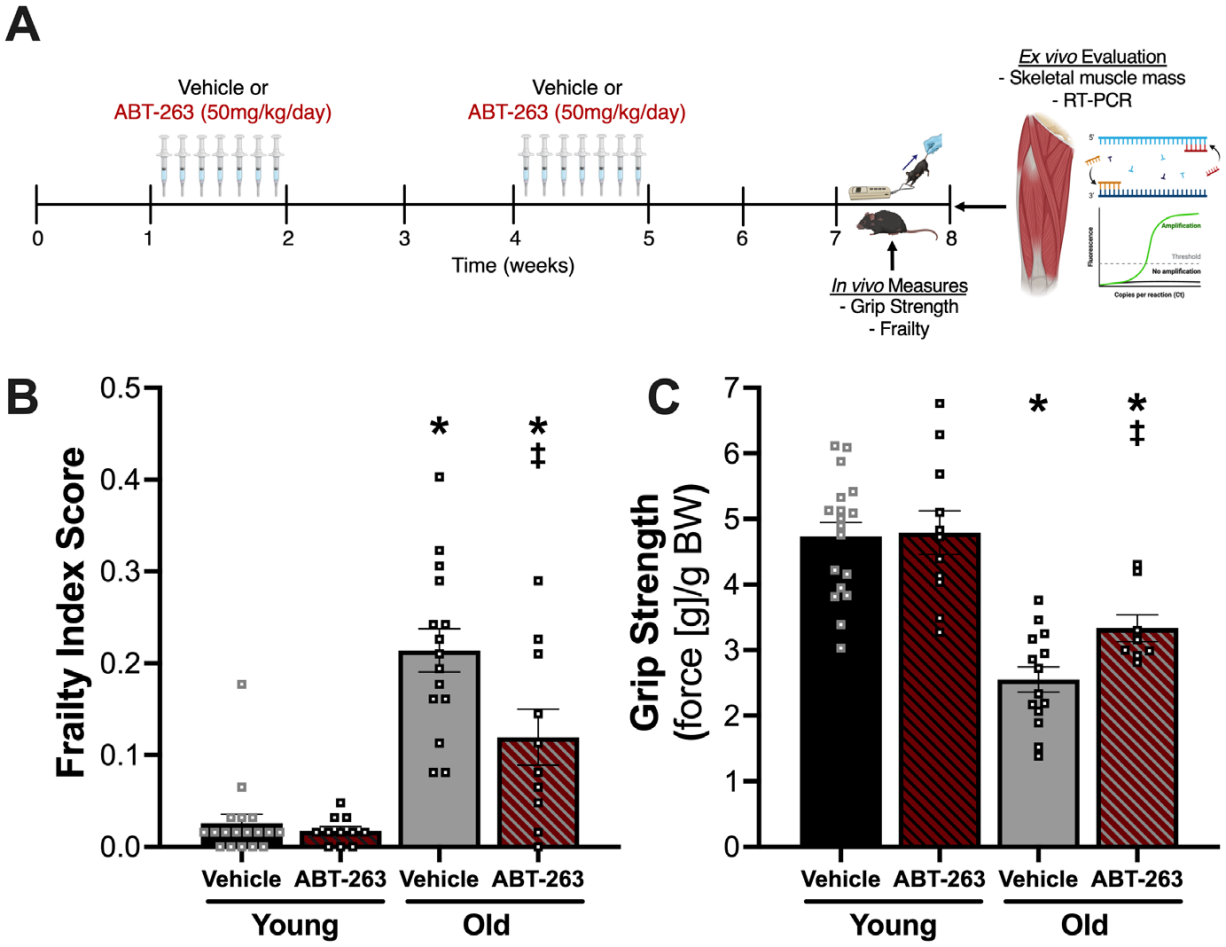
Oral senolytic therapy with the synthetic pharmacological agent ABT‐263 lessens the age‐related differences in frailty index score and grip strength in young and old mice. Experimental design (A). Frailty index score (B) and normalized grip strength (C). Squares = male mice, inverted triangles = female mice. Data presented as mean ± SEM. One‐way ANOVA corrected with Tukey's multiple comparisons test. *p* < 0.05: *both young groups; ^‡^old vehicle. Images made with Biorender.com.

Old mice treated with the vehicle (Figure 5A) had a higher frailty index score (0.22 ± 0.02 AU, *N* = 15) (Figure 5B; Table S3) and lower grip strength (2.55 ± 0.19 g/g BW, *N* = 14) (Figure 5C) compared with young Veh‐treated mice (*N* = 18) (0.03 ± 0.01 AU; 4.74 ± 0.21 g/g BW; both *p* < 0.0001). Old mice orally administered ABT‐263 had a 44% lower frailty index score (0.112 ± 0.03 AU, *N* = 10; *p* = 0.0069) (Figure 5B; Table S3) and a 23% higher grip strength (3.34 ± 0.21 g/g BW, *N* = 8; *p* = 0.0172) (Figure 5C) compared with old Veh‐treated mice. Old mice treated with the vehicle had higher expression of *Cdkn1a* (Figure S3A & Data S1) and *Cdkn2a* (Figure S3B & Data S1) compared with young Veh‐treated mice (*N* = 8‐10/group; *Cdkn1a*: *p* = 0.0001, *Cdkn2a*: *p* < 0.0001). Oral senolytic therapy with ABT‐263 (Figure 5A) favorably altered the age‐associated increase in frailty index score (Figure 5B; Table S3) and decrease in grip strength (Figure 5C) by 44% and 34%, respectively. Additionally, old mice treated with ABT‐263 (*N* = 9–10) had a 76% lower expression of *Cdkn1a* (*p* < 0.0001; Figure S3A & Data S1) and a 45% lower expression of *Cdkn2a* (*p* = 0.0057; Figure S3B & Data S1) compared with old mice treated with the vehicle. Expression levels of *Cdkn1a* (*p* = 0.9997) and *Cdkn2a* (*p* = 0.1868) in the skeletal muscle of old ABT‐263‐treated mice were similar to that observed in young mice (Figure S3A,B & Data S1).

Oral senolytic therapy with ABT‐263 did not alter frailty index score (0.02 ± 0.01 AU; *p* = 0.9881 vs. Young Veh) (Figure 5B; Table S3) or grip strength (4.79 ± 0.33 g/g BW; *p* = 0.9982 vs. Young Veh) (Figure 5C) in young mice (*N* = 11). Similar to studies in GCV‐treated p16‐3MR mice, there was no effect of age or treatment on *Pai1* (Figure S3C & Data S1) or *Lmnb1* (Figure S3D & Data S1) gene expression in skeletal muscle (*N* = 9‐10/group).

Fisetin improves physical function to a similar extent as the genetic‐based clearance of excess p16+ senescent cells and oral synthetic senolytic therapy with ABT‐263 in old mice.

Upon completion of the two additional independent studies, we then compared the improvements in frailty index score and grip strength with fisetin in old mice to those observed following genetic‐based clearance of senescent cells and oral intermittent supplementation with the synthetic senolytic ABT‐263. The comparisons were conducted using each of the frailty index score and grip strength outcomes made relative to the Old Vehicle‐treated group in each respective study.

**FIGURE 6:**
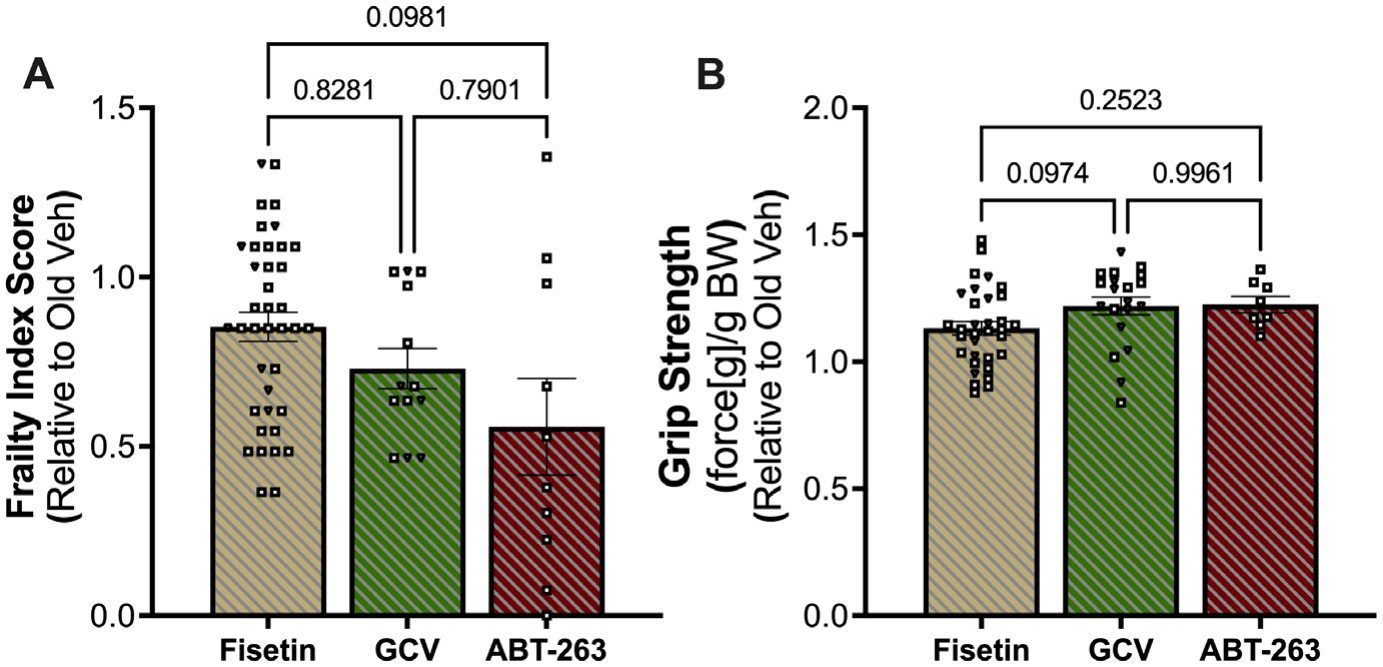
Comparison of efficacy for improvements in frailty index score and grip strength. Frailty index score (A) and normalized grip strength (B). Squares = male mice, inverted triangles = female mice. Data are relative to old vehicle‐treated mice in each respective study. Data presented as mean ± SEM. One‐way ANOVA corrected with Tukey's multiple comparisons test.

Frailty index score was similarly lower in Old Fisetin‐treated mice (0.85 ± 0.04) as compared with Old GCV‐treated (0.73 ± 0.06; *p* = 0.8281) and Old ABT‐263‐treated (0.56 ± 0.14; *p* = 0.0981) mice (Figure 6A). There were no differences observed in the magnitude of reduction in frailty index score between Old GCV‐treated and Old ABT‐263‐treated mice (*p* = 0.7901) (Figure 6A).

Grip strength was similarly higher in Old Fisetin‐treated mice (1.14 ± 0.03) when compared with Old GCV‐treated (1.22 ± 0.04; *p* = 0.0974) and Old ABT‐263‐treated (1.23 ± 0.03; *p* = 0.2523) mice (Figure 6B). There were no differences observed in the extent of improvement in grip strength between Old GCV‐treated and Old ABT‐263‐treated mice (*p* = 0.9961) (Figure 6B).

## 4 Discussion

In the present study, we demonstrated the efficacy of oral intermittent supplementation with the natural senolytic fisetin for attenuating the age‐related increase in frailty and decrease in skeletal muscle strength in male and female mice. The improvements in frailty and skeletal muscle strength with fisetin treatment were accompanied by an amelioration of age‐associated shifts in the skeletal muscle transcriptome and beneficial modulation of gene expression in old mice, including reductions in cellular senescence and cell senescence‐related genes. We also compared the relative effectiveness of fisetin on frailty and grip strength to two other well‐established approaches for clearing excess senescent cells. We found that fisetin improves frailty and grip strength with aging to a similar extent as genetic clearance of excess p16+ senescent cells and oral intermittent treatment with the synthetic senolytic ABT‐263. Taken together, our findings suggest that treatment with fisetin may be a viable therapeutic approach to improve physical function and reduce excess cell senescence in skeletal muscle with aging.

Declines in physical function with aging are antecedent to clinical disease and disability (Celis‐Morales et al. 2018; Court et al. 2025; Grevendonk et al. 2021; Manning et al. 2024). Cellular senescence is an emerging driver of age‐related physical dysfunction, but effective senolytic approaches for improving domains of physical function with aging remain to be established. To address the need for senolytic compounds with greater translational potential for administration in healthy older humans, we sought to obtain preclinical proof‐of‐concept evidence of the efficacy of oral intermittent senolytic therapy with the flavonoid fisetin to reduce frailty and improve grip strength. We observed a reduction of the age‐associated increase in frailty with fisetin treatment using a frailty index that is reflective of clinical frailty indices in humans (Whitehead et al. 2014). The improvement in the overall frailty index score following fisetin treatment was a result of a collection of subtle changes across the frailty index, suggesting effects of fisetin on multiple physiological systems. We also found that fisetin treatment in old mice improved grip strength with aging, which is a strong predictor of the risk of chronic disease and all‐cause mortality in humans (Bohannon 2019; Celis‐Morales et al. 2018). The magnitude of the effect of fisetin treatment on grip strength in old mice is similar to that observed in other studies using synthetic senolytic treatment (Xu et al. 2018) or genetic‐based clearance of senescent cells (Wang et al. 2024). These findings extend ours and others' observations of beneficial effects of fisetin on age‐related physiological dysfunction (Mahoney et al. 2024; Tavenier et al. 2024; Yousefzadeh et al. 2018) and support fisetin as a novel senolytic approach for enhancing physical function with advancing age.

To gain insight into the cellular senescence‐associated signaling pathways that may be mechanistically linked with the effects of fisetin on physical function, we performed an in‐depth molecular characterization of skeletal muscle using transcriptomics. We focused on the patterns of gene expression that were most affected by aging and fisetin treatment. Overall, we observed that fisetin attenuated many broad shifts in gene expression that were observed in Old Veh mice relative to Young Veh mice. Notably, many of these effects were driven by differences in cell senescence signaling as we found that the *cellular senescence* and *p53 signaling* KEGG pathways were no longer enriched in old mice after fisetin treatment. An amelioration of the age‐related increase in the fundamental biomarker of cellular senescence *Cdkn1a* (Englund et al. 2023; Perez et al. 2022; Zhang et al. 2022) (i.e., p21) in skeletal muscle appeared to be a key event responsible for the observed effects of fisetin on cell senescence. Complementary findings in D‐galactose‐induced senescent C2C12 myoblasts in vitro also support fisetin as a senolytic in skeletal muscle‐residing cells (Zhang et al. 2025). Fisetin also attenuated age‐associated differences in other senescent‐ and SASP‐related genes, including genes involved in MAPK signaling, growth arrest, and DNA damage (Anerillas et al. 2020; Englund et al. 2023; Novais et al. 2021). Senescent‐ and SASP‐associated genes upregulated with fisetin also serve as key regulators of senescent cell burden (He et al. 2022) and skeletal muscle repair, regeneration, and hypertrophy signaling (Deng et al. 2021; Khandelwal et al. 2011; Sasako et al. 2022; Tsai et al. 2015; Weber and Carroll 2021).

We also observed that the expression of *Ddit4* (DNA damage‐inducible transcript 4 or *Redd1*) was lower in the skeletal muscle of old mice treated with fisetin compared with vehicle. *Ddit4* induces cellular senescence in part by upregulating *Cdkn1a* (Hulmi et al. 2018) and is an inhibitor of mTORC1, a protein complex that regulates skeletal muscle hypertrophy (Coronel et al. 2022; Foltyn et al. 2019). Also, *Ddit4* gene expression is reduced in the skeletal muscle of old mice after intermittent administration of the synthetic senolytic dasatinib + quercetin (Dungan et al. 2022a). Taken together, it is possible that the effects of fisetin on cell senescence‐related signaling pathways may improve the microenvironment in aged skeletal muscle to enhance repair and regeneration processes to reduce frailty and improve skeletal muscle strength.

To build on our findings that fisetin treatment reduces the age‐related increase in *Cdkn1a* expression and decreases *Ddit4* expression in the skeletal muscle of old mice, we conducted a retrospective deconvolution analysis to identify which cell types in skeletal muscle are responsible for the beneficial effects of fisetin (Chu et al. 2022). The results from this analysis may indicate that the consequences of fisetin treatment on cell senescence‐related gene expression occur in several cell types, including differentiated (e.g., myonuclei) and stem cells (e.g., fibro‐adipogenic progenitors). Together, these findings suggest lower frailty and higher grip strength may be the result of the effects of fisetin in many skeletal muscle cell types that contribute to force production and overall function.

After showing the efficacy of fisetin on indices of physical function and determining the senolytic effects in skeletal muscle, we conducted two additional intervention studies with the primary goal of determining how the effects of fisetin on frailty and physical function compare with more targeted senolytic approaches. We first evaluated the effects of genetic‐based clearance of excess p16+ senescent cells (Clayton et al. 2023; Demaria et al. 2014) and found that genetic elimination lessened the age‐related changes in frailty and grip strength. Of note, although genetic‐based clearance of p21+ senescent cells has been shown to improve frailty and indices of physical function in old mice (Wang et al. 2024), our observations are the first to show that selectively eliminating p16+ cells improves frailty and physical function with aging. Importantly, the degree of improvement with p16+ senescent cell clearance was not statistically different from that observed with fisetin, suggesting that fisetin is at least as effective.

Lastly, we assessed the efficacy of oral senolytic therapy with the synthetic pharmacological agent ABT‐263. We found that ABT‐263 diminished the age‐associated changes in frailty index score and grip strength, which were accompanied by lower gene expression of the senescent cell markers p16 and p21 in skeletal muscle. These observations are the first to show effects of ABT‐263 on a comprehensive clinically relevant frailty index and extend previous findings of improvements in grip strength in old mice with the synthetic senolytic drug dasatinib and quercetin combination (Xu et al. 2018). We found that the beneficial changes in frailty and grip strength with fisetin treatment were to a similar extent as observed with ABT‐263.

Although the effects of GCV‐mediated senolysis and ABT‐263 treatment on physical function were not statistically different from the effects of fisetin, there was some indication of possibly greater influence of clearance of genetic elimination of p16+ cells and ABT‐263 on physical function. This may have been related to the effects of GCV in the p16‐3MR mouse and ABT‐263 on p16+ senescent cells, as effects on *Cdkn2a* (i.e., p16) were not observed with fisetin. Regardless, these findings advance fisetin as a natural food‐derived therapeutic strategy for clinical application to target excess senescent cells in skeletal muscle and improve physical function with primary aging.

In this study, our data using RNA sequencing support the notion that fisetin is a senolytic in skeletal muscle; however, senolytic effects observed via gene expression are generally confirmed with cell experiments or histological evaluation. Complementary work demonstrating the senolytic effect of fisetin on D‐galactose‐induced senescent myoblasts was recently performed (Zhang et al. 2025). However, additional research is needed to more comprehensively establish the senolytic and/or senomorphic effects of fisetin in skeletal muscle, including the primary cell types affected in the context of aging.

## 5 Conclusions

Here, we demonstrate efficacy for targeting cellular senescence through oral intermittent supplementation with fisetin, a natural senolytic compound with high translational potential for administration in humans, to mitigate frailty and reductions in grip strength with aging. We also provide novel insight into the cell senescence‐related signaling pathways modulated by fisetin in skeletal muscle that may contribute to the beneficial effects of fisetin on physical function. Lastly, we show that fisetin improved physical function to a similar degree as genetic‐based clearance of senescent cells and oral intermittent supplementation with the synthetic senolytic ABT‐263. As such, our preclinical findings provide the necessary proof‐of‐concept evidence to support the use of intermittent fisetin supplementation as a treatment for improving frailty and skeletal muscle strength with aging to potentially prevent functional limitations and a loss of independence.

## Author Contributions

Z.S.C., M.J.R., S.A.M., and D.R.S. designed the initial study; data were acquired by Z.S.C., K.R.L., N.S.V., J.H.M.‐D., and K.O.M.; data were analyzed by K.O.M. and Z.S.C.; data were interpreted by K.O.M., R.T.M., M.J.R., and Z.S.C.; K.O.M. created tables/figures; K.O.M. and Z.S.C. wrote the initial draft; and all authors edited and approved the final draft.

## Disclosure

The authors have nothing to report.

## Conflicts of Interest

The authors declare no conflicts of interest.

## Supporting information


Table S1.

Table S2.

Table S3.

Figures S1–S3.

Data S1.

Data S2.

Data S3.

## Data Availability

The data that supports the findings of this study are available in the Supporting Information of this article. Supporting Information, including raw PCR values, fold change and statistical values for bulk RNAseq and deconvolution analyses, and frailty tables for each study, can be found on a FigShare repository https://doi.org/10.6084/m9.figshare.26520916.
