# Supplementary material for: Intermittent Supplementation With Fisetin Improves Physical Function and Decreases Cellular Senescence in Skeletal Muscle With Aging: A Comparison to Genetic Clearance of Senescent Cells and Synthetic Senolytic Approaches
Source: Aging Cell. 2025 May 28;24(8):e70114. doi: 10.1111/acel.70114 (PMC12341784; doi:10.1111/acel.70114)
Supplement: Supplementary file 1 — Table S1. [file ACEL-24-e70114-s007.docx]

Table S1. Summed scores of individual frailty domains and subindices with aging and/or fisetin treatment.

| *Frailty Domain* | Y-Veh | Y-Fisetin | O-Veh | O-Fisetin |
| --- | --- | --- | --- | --- |
| Integument | 0.04 ± 0.04 | 0.04 ± 0.04 | 1.47 ± 0.19* | 1.38 ± 0.27* |
| *Alopecia* | 0.04 ± 0.04 | 0.00 ± 0.00 | 0.49 ± 0.14* | 0.40 ± 0.05* |
| *Loss of fur color* | 0.00 ± 0.00 | 0.00 ± 0.00 | 0.47 ± 0.04* | 0.43 ± 0.04* |
| *Dermatitis* | 0.00 ± 0.00 | 0.00 ± 0.00 | 0.04 ± 0.02 | 0.05 ± 0.04* |
| *Loss of whiskers* | 0.00 ± 0.00 | 0.00 ± 0.00 | 0.00 ± 0.00 | 0.06 ± 0.04* |
| *Coat condition* | 0.00 ± 0.00 | 0.04 ± 0.04 | 0.44 ± 0.05* | 0.44 ± 0.05* |
| Physical/Musculoskeletal | 0.14 ± 0.11 | 0.27 ± 0.19 | 3.21 ± 0.37* | 3.15 ± 0.35* |
| *Tumors* | 0.02 ± 0.02 | 0.15 ± 0.09 | 0.21 ± 0.07* | 0.33 ± 0.07* |
| *Distended abdomen* | 0.00 ± 0.00 | 0.00 ± 0.00 | 0.19 ± 0.04 | 0.16 ± 0.04* |
| *Kyphosis* | 0.00 ± 0.00 | 0.00 ± 0.00 | 0.50 ± 0.03* | 0.48 ± 0.03* |
| *Tail stiffening* | 0.08 ± 0.05 | 0.08 ± 0.06 | 0.87 ± 0.04* | 0.83 ± 0.05* |
| *Gait disorders* | 0.00 ± 0.00 | 0.00 ± 0.00 | 0.39 ± 0.05* | 0.39 ± 0.04* |
| *Tremor* | 0.00 ± 0.00 | 0.00 ± 0.00 | 0.50 ± 0.04* | 0.49 ± 0.03* |
| *Forelimb grip strength* | 0.04 ± 0.04 | 0.04 ± 0.04 | 0.16 ± 0.04* | 0.09 ± 0.05*^‡^ |
| *Body condition score* | 0.00 ± 0.00 | 0.00 ± 0.00 | 0.39 ± 0.05* | 0.38 ± 0.04* |
| Vestibulocochlear/Auditory | 0.00 ± 0.00 | 0.00 ± 0.00 | 1.47 ± 0.15* | 1.40 ± 0.11* |
| *Vestibular disturbance* | 0.00 ± 0.00 | 0.00 ± 0.00 | 0.54 ± 0.08* | 0.51 ± 0.07* |
| *Hearing loss* | 0.00 ± 0.00 | 0.00 ± 0.00 | 0.93 ± 0.07* | 0.89 ± 0.04* |
| Ocular/Nasal | 0.08 ± 0.08 | 0.04 ± 0.01 | 1.33 ± 0.25* | 1.28 ± 0.27* |
| *Cataracts* | 0.00 ± 0.00 | 0.00 ± 0.00 | 0.21 ± 0.08* | 0.13 ± 0.05* |
| *Corneal opacity* | 0.04 ± 0.04 | 0.00 ± 0.00 | 0.09 ± 0.03 | 0.05 ± 0.03 |
| *Eye discharge/swelling* | 0.00 ± 0.00 | 0.00 ± 0.00 | 0.13 ± 0.04 | 0.18 ± 0.05 |
| *Microphthalmia* | 0.00 ± 0.00 | 0.00 ± 0.00 | 0.00 ± 0.00 | 0.01 ± 0.03*^‡^ |
| *Vision loss* | 0.04 ± 0.04 | 0.00 ± 0.00 | 0.83 ± 0.05* | 0.81 ± 0.05* |
| *Menace reflex* | 0.00 ± 0.00 | 0.04 ± 0.04 | 0.06 ± 0.04* | 0.08 ± 0.04* |
| *Nasal discharge* | 0.00 ± 0.00 | 0.00 ± 0.00 | 0.01 ± 0.01 | 0.02 ± 0.03* |
| Digestive/Urogenital | 0.04 ± 0.04 | 0.00 ± 0.00 | 0.39 ± 0.12* | 0.30 ± 0.09* |
| *Malocclusions* | 0.00 ± 0.00 | 0.00 ± 0.00 | 0.06 ± 0.04* | 0.02 ± 0.02* |
| *Rectal prolapse* | 0.00 ± 0.00 | 0.00 ± 0.00 | 0.09 ± 0.04* | 0.10 ± 0.03* |
| *Vaginal/uterine/penile prolapse* | 0.04 ± 0.04 | 0.00 ± 0.00 | 0.24 ± 0.04* | 0.18 ± 0.04* |
| *Diarrhea* | 0.00 ± 0.00 | 0.00 ± 0.00 | 0.00 ± 0.00 | 0.00 ± 0.00 |
| Respiratory (breathing rate) | 0.00 ± 0.00 | 0.00 ± 0.00 | 0.00 ± 0.00 | 0.00 ± 0.00 |
| Discomfort | 0.00 ± 0.00 | 0.00 ± 0.00 | 0.01 ± 0.01 | 0.00 ± 0.00 |
| *Mouse grimace scale* | 0.00 ± 0.00 | 0.00 ± 0.00 | 0.01 ± 0.01 | 0.00 ± 0.00 |
| *Piloerection* | 0.00 ± 0.00 | 0.00 ± 0.00 | 0.00 ± 0.00 | 0.00 ± 0.00 |
| Temperature | 0.00 ± 0.00 | 0.00 ± 0.00 | 0.04 ± 0.04* | 0.00 ± 0.00*^‡^ |
| Body Weight | 0.11 ± 0.06 | 0.13 ± 0.09 | 0.11 ± 0.08 | 0.02 ± 0.02*^‡^ |

Data are mean ± SEM. **P* < 0.05 between ages within group. ^‡^*P* < 0.05 between groups within age.
