# Supplementary material for: Intermittent Supplementation With Fisetin Improves Physical Function and Decreases Cellular Senescence in Skeletal Muscle With Aging: A Comparison to Genetic Clearance of Senescent Cells and Synthetic Senolytic Approaches
Source: Aging Cell. 2025 May 28;24(8):e70114. doi: 10.1111/acel.70114 (PMC12341784; doi:10.1111/acel.70114)
Supplement: Supplementary file 2 — Table S2. [file ACEL-24-e70114-s003.docx]

Table S2. Summed scores of individual frailty domains and subindices with aging and/or ganciclovir (GCV) treatment.

| *Frailty Domain* | Y-Veh | Y-GCV | O-Veh | O-GCV |
| --- | --- | --- | --- | --- |
| Integument | 0.13 ± 0.05 | 0.15 ± 0.06 | 1.44 ± 0.29* | 1.09 ± 0.27* |
| *Alopecia* | 0.00 ± 0.00 | 0.00 ± 0.00 | 0.43 ± 0.08* | 0.34 ± 0.07* |
| *Loss of fur color* | 0.00 ± 0.00 | 0.09 ± 0.06 | 0.36 ± 0.06* | 0.24 ± 0.05* |
| *Dermatitis* | 0.00 ± 0.00 | 0.00 ± 0.00 | 0.12 ± 0.06 | 0.16 ± 0.07* |
| *Loss of whiskers* | 0.00 ± 0.00 | 0.00 ± 0.00 | 0.05 ± 0.03* | 0.03 ± 0.02* |
| *Coat condition* | 0.13 ± 0.05 | 0.15 ± 0.06 | 0.48 ± 0.06* | 0.32 ± 0.06* |
| Physical/Musculoskeletal | 0.19 ± 0.11 | 0.23 ± 0.14 | 3.16 ± 0.52* | 2.30 ± 0.24* |
| *Tumors* | 0.03 ± 0.03 | 0.04 ± 0.03 | 0.40 ± 0.11* | 0.45 ± 0.11* |
| *Distended abdomen* | 0.00 ± 0.00 | 0.00 ± 0.00 | 0.05 ± 0.04 | 0.00 ± 0.00* |
| *Kyphosis* | 0.00 ± 0.00 | 0.00 ± 0.00 | 0.45 ± 0.06* | 0.34 ± 0.06* |
| *Tail stiffening* | 0.03 ± 0.03 | 0.04 ± 0.03 | 0.86 ± 0.05* | 0.71 ± 0.05* |
| *Gait disorders* | 0.00 ± 0.00 | 0.00 ± 0.00 | 0.40 ± 0.07* | 0.32 ± 0.05* |
| *Tremor* | 0.13 ± 0.05 | 0.11 ± 0.05 | 0.24 ± 0.06* | 0.29 ± 0.06* |
| *Forelimb grip strength* | 0.00 ± 0.00 | 0.00 ± 0.00 | 0.31 ± 0.09* | 0.05 ± 0.05*^‡^ |
| *Body condition score* | 0.00 ± 0.00 | 0.04 ± 0.03 | 0.45 ± 0.06* | 0.34 ± 0.06* |
| Vestibulocochlear/Auditory | 0.00 ± 0.00 | 0.04 ± 0.03 | 1.17 ± 0.22* | 0.95 ± 0.19* |
| *Vestibular disturbance* | 0.00 ± 0.00 | 0.04 ± 0.03 | 0.50 ± 0.11* | 0.42 ± 0.10* |
| *Hearing loss* | 0.00 ± 0.030 | 0.00 ± 0.00 | 0.67 ± 0.11* | 0.58 ± 0.11* |
| Ocular/Nasal | 0.00 ± 0.00 | 0.08 ± 0.06 | 1.19 ± 0.22* | 0.72 ± 0.23* |
| *Cataracts* | 0.00 ± 0.00 | 0.00 ± 0.00 | 0.26 ± 0.08* | 0.24 ± 0.07* |
| *Corneal opacity* | 0.00 ± 0.00 | 0.04 ± 0.03 | 0.17 ± 0.08* | 0.03 ± 0.02 |
| *Eye discharge/swelling* | 0.00 ± 0.00 | 0.00 ± 0.00 | 0.14 ± 0.07* | 0.03 ± 0.03 |
| *Microphthalmia* | 0.00 ± 0.00 | 0.04 ± 0.03 | 0.05 ± 0.05* | 0.00 ± 0.00*^‡^ |
| *Vision loss* | 0.00 ± 0.00 | 0.00 ± 0.00 | 0.55 ± 0.10* | 0.42 ± 0.11* |
| *Menace reflex* | 0.00 ± 0.00 | 0.00 ± 0.00 | 0.00 ± 0.00 | 0.00 ± 0.00 |
| *Nasal discharge* | 0.00 ± 0.00 | 0.00 ± 0.00 | 0.02 ± 0.02* | 0.00 ± 0.00 |
| Digestive/Urogenital | 0.00 ± 0.00 | 0.00 ± 0.00 | 0.38 ± 0.09* | 0.05 ± 0.03*^‡^ |
| *Malocclusions* | 0.00 ± 0.00 | 0.00 ± 0.00 | 0.12 ± 0.07* | 0.00 ± 0.00* |
| *Rectal prolapse* | 0.00 ± 0.00 | 0.00 ± 0.00 | 0.17 ± 0.07* | 0.00 ± 0.00 |
| *Vaginal/uterine/penile prolapse* | 0.00 ± 0.00 | 0.00 ± 0.00 | 0.14 ± 0.05* | 0.05 ± 0.03 |
| *Diarrhea* | 0.00 ± 0.00 | 0.00 ± 0.00 | 0.00 ± 0.00 | 0.00 ± 0.00 |
| Respiratory (breathing rate) | 0.00 ± 0.00 | 0.00 ± 0.00 | 0.04 ± 0.04* | 0.00 ± 0.00 |
| Discomfort | 0.00 ± 0.00 | 0.00 ± 0.00 | 0.04 ± 0.04* | 0.00 ± 0.00 |
| *Mouse grimace scale* | 0.00 ± 0.00 | 0.00 ± 0.00 | 0.05 ± 0.05* | 0.00 ± 0.00 |
| *Piloerection* | 0.00 ± 0.00 | 0.00 ± 0.00 | 0.00 ± 0.00 | 0.00 ± 0.00 |
| Temperature | 0.00 ± 0.00 | 0.00 ± 0.00 | 0.10 ± 0.04* | 0.07 ± 0.04* |
| Body Weight | 0.03 ± 0.03 | 0.08 ± 0.05 | 0.10 ± 0.05* | 0.12 ± 0.05 |

Data are mean ± SEM. **P* < 0.05 between ages within group. ^‡^*P* < 0.05 between groups within age.
