# Supplementary material for: Intermittent Supplementation With Fisetin Improves Physical Function and Decreases Cellular Senescence in Skeletal Muscle With Aging: A Comparison to Genetic Clearance of Senescent Cells and Synthetic Senolytic Approaches
Source: Aging Cell. 2025 May 28;24(8):e70114. doi: 10.1111/acel.70114 (PMC12341784; doi:10.1111/acel.70114)
Supplement: Supplementary file 3 — Table S3. [file ACEL-24-e70114-s002.docx]

Table S3. Summed scores of individual frailty domains and subindices with aging and/or ABT-263 treatment.

| *Frailty Domain* | Y-Veh | Y-ABT263 | O-Veh | O-ABT263 |
| --- | --- | --- | --- | --- |
| Integument | 0.27 ± 0.12 | 0.27 ± 0.02 | 1.58 ± 0.23* | 1.05 ± 0.36* |
| *Alopecia* | 0.03 ± 0.03 | 0.00 ± 0.00 | 0.43 ± 0.09* | 0.25 ± 0.08* |
| *Loss of fur color* | 0.16 ± 0.04 | 0.09 ± 0.06 | 0.54 ± 0.03* | 0.35 ± 0.8* |
| *Dermatitis* | 0.00 ± 0.00 | 0.00 ± 0.00 | 0.00 ± 0.00 | 0.05 ± 0.05* |
| *Loss of whiskers* | 0.00 ± 0.00 | 0.00 ± 0.00 | 0.07 ± 0.05* | 0.05 ± 0.05* |
| *Coat condition* | 0.08 ± 0.05 | 0.18 ± 0.08 | 0.54 ± 0.06* | 0.35 ± 0.10* |
| Physical/Musculoskeletal | 0.46 ± 0.34 | 0.14 ± 0.01 | 3.26 ± 0.44* | 2.55 ± 0.91* |
| *Tumors* | 0.06 ± 0.06 | 0.05 ± 0.05 | 0.50 ± 0.16* | 0.40 ± 0.16* |
| *Distended abdomen* | 0.06 ± 0.04 | 0.00 ± 0.00 | 0.00 ± 0.00 | 0.00 ± 0.00* |
| *Kyphosis* | 0.03 ± 0.03 | 0.00 ± 0.00 | 0.61 ± 0.09* | 0.40 ± 0.12*^‡^ |
| *Tail stiffening* | 0.11 ± 0.05 | 0.05 ± 0.05 | 0.82 ± 0.06* | 0.75 ± 0.08* |
| *Gait disorders* | 0.03 ± 0.03 | 0.00 ± 0.00 | 0.29 ± 0.10* | 0.20 ± 0.15* |
| *Tremor* | 0.06 ± 0.04 | 0.00 ± 0.00 | 0.46 ± 0.08* | 0.40 ± 0.12* |
| *Forelimb grip strength* | 0.08 ± 0.06 | 0.00 ± 0.00 | 0.29 ± 0.07* | 0.10 ± 0.05*^‡^ |
| *Body condition score* | 0.03 ± 0.03 | 0.05 ± 0.05 | 0.29 ± 0.10* | 0.30 ± 0.13* |
| Vestibulocochlear/Auditory | 0.03 ± 0.03 | 0.00 ± 0.00 | 0.79 ± 0.26* | 0.55 ± 0.25* |
| *Vestibular disturbance* | 0.00 ± 0.00 | 0.00 ± 0.00 | 0.36 ± 0.13* | 0.15 ± 0.10* |
| *Hearing loss* | 0.03 ± 0.03 | 0.00 ± 0.00 | 0.43 ± 0.13* | 0.40 ± 0.16* |
| Ocular/Nasal | 0.08 ± 0.01 | 0.05 ± 0.05 | 0.75 ± 0.26* | 0.60 ± 0.32* |
| *Cataracts* | 0.00 ± 0.00 | 0.00 ± 0.00 | 0.18 ± 0.06* | 0.10 ± 0.06* |
| *Corneal opacity* | 0.00 ± 0.00 | 0.00 ± 0.00 | 0.00 ± 0.00 | 0.05 ± 0.05 |
| *Eye discharge/swelling* | 0.03 ± 0.03 | 0.05 ± 0.05 | 0.00 ± 0.00 | 0.05 ± 0.05 |
| *Microphthalmia* | 0.00 ± 0.00 | 0.00 ± 0.00 | 0.07 ± 0.07 | 0.00 ± 0.00*^‡^ |
| *Vision loss* | 0.06 ± 0.06 | 0.00 ± 0.00 | 0.50 ± 0.13* | 0.40 ± 0.16* |
| *Menace reflex* | 0.00 ± 0.00 | 0.00 ± 0.00 | 0.00 ± 0.00 | 0.00 ± 0.00 |
| *Nasal discharge* | 0.00 ± 0.00 | 0.00 ± 0.00 | 0.00 ± 0.00 | 0.00 ± 0.00 |
| Digestive/Urogenital | 0.03 ± 0.03 | 0.00 ± 0.00 | 0.57 ± 0.28* | 0.05 ± 0.05^‡^ |
| *Malocclusions* | 0.00 ± 0.00 | 0.00 ± 0.00 | 0.32 ± 0.13* | 0.05 ± 0.05* |
| *Rectal prolapse* | 0.00 ± 0.00 | 0.00 ± 0.00 | 0.07 ± 0.07* | 0.00 ± 0.00 |
| *Vaginal/uterine/penile prolapse* | 0.03 ± 0.03 | 0.00 ± 0.00 | 0.18 ± 0.08* | 0.00 ± 0.00 |
| *Diarrhea* | 0.00 ± 0.00 | 0.00 ± 0.00 | 0.00 ± 0.00 | 0.00 ± 0.00 |
| Respiratory (breathing rate) | 0.03 ± 0.01 | 0.00 ± 0.00 | 0.00 ± 0.00 | 0.00 ± 0.00 |
| Discomfort | 0.00 ± 0.00 | 0.00 ± 0.00 | 0.00 ± 0.00* | 0.00 ± 0.00 |
| *Mouse grimace scale* | 0.00 ± 0.00 | 0.00 ± 0.00 | 0.00 ± 0.00 | 0.00 ± 0.00 |
| *Piloerection* | 0.00 ± 0.00 | 0.00 ± 0.00 | 0.00 ± 0.00 | 0.00 ± 0.00 |
| Temperature | 0.00 ± 0.00 | 0.00 ± 0.00 | 0.04 ± 0.04* | 0.00 ± 0.00^‡^ |
| Body Weight | 0.06 ± 0.01 | 0.09 ± 0.06 | 0.11 ± 0.08* | 0.15 ± 0.11* |

Data are mean ± SEM. **P* < 0.05 vs. Y-Veh; ^‡^*P* < 0.05 vs. O-Veh
