# Supplementary material for: Intermittent Supplementation With Fisetin Improves Physical Function and Decreases Cellular Senescence in Skeletal Muscle With Aging: A Comparison to Genetic Clearance of Senescent Cells and Synthetic Senolytic Approaches
Source: Aging Cell. 2025 May 28;24(8):e70114. doi: 10.1111/acel.70114 (PMC12341784; doi:10.1111/acel.70114)
Supplement: Supplementary file 4 — Figures S1–S3. [file ACEL-24-e70114-s005.pptx]

## Slide 1
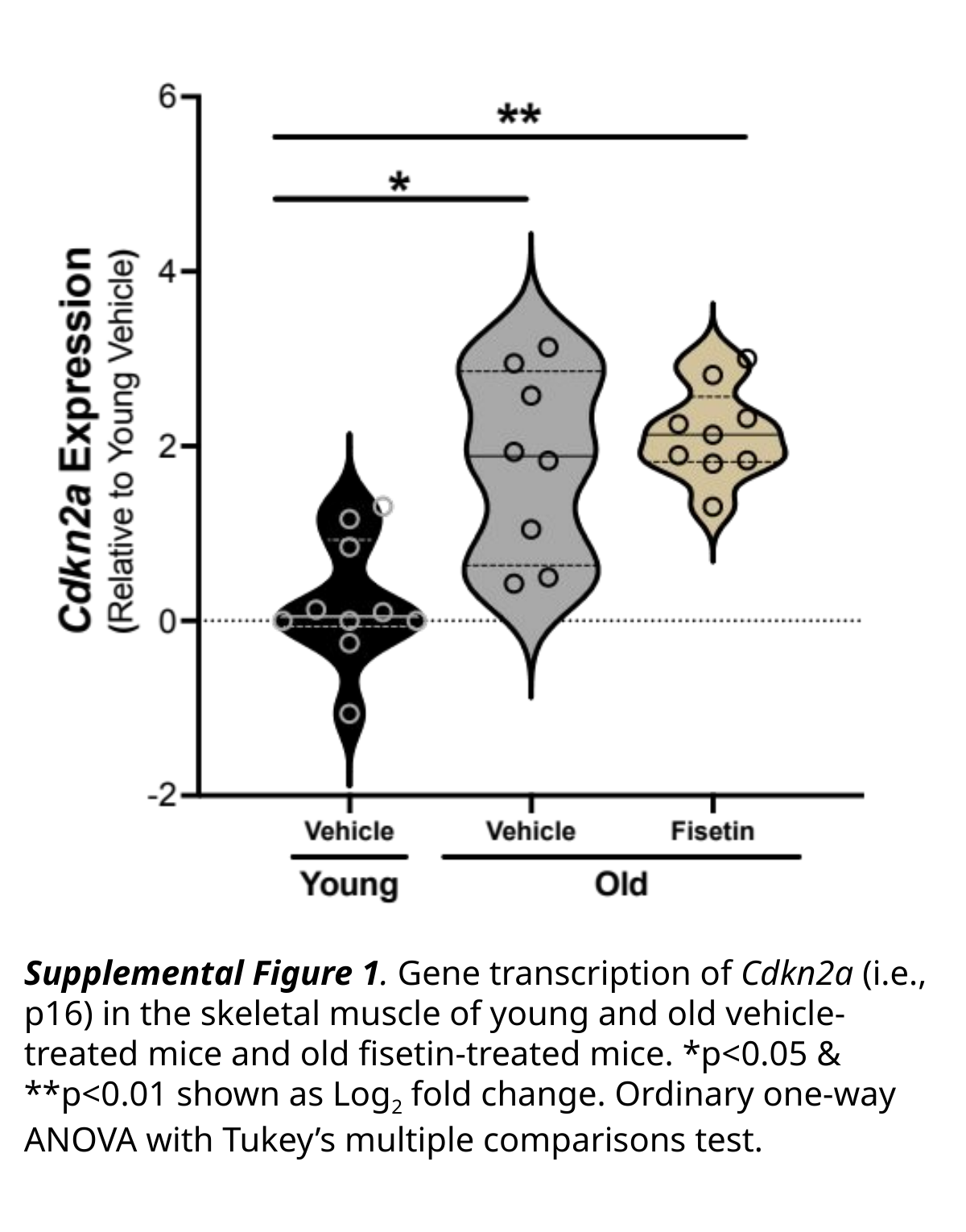

Supplemental Figure 1. Gene transcription of Cdkn2a (i.e., p16) in the skeletal muscle of young and old vehicle-treated mice and old fisetin-treated mice. *p<0.05 & **p<0.01 shown as Log2 fold change. Ordinary one-way ANOVA with Tukey’s multiple comparisons test.

## Slide 2
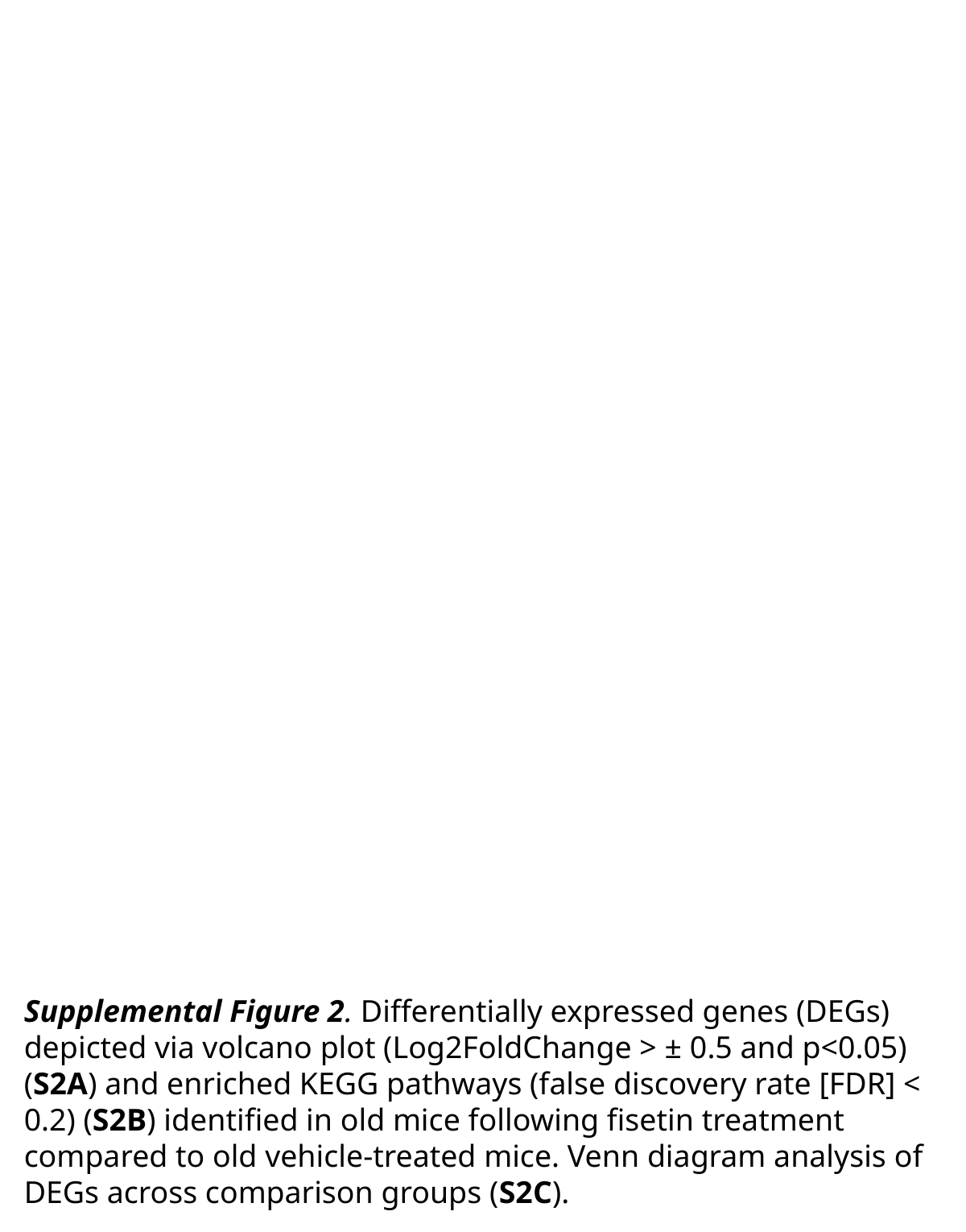

Supplemental Figure 2. Differentially expressed genes (DEGs) depicted via volcano plot (Log2FoldChange > ± 0.5 and p<0.05) (S2A) and enriched KEGG pathways (false discovery rate [FDR] < 0.2) (S2B) identified in old mice following fisetin treatment compared to old vehicle-treated mice. Venn diagram analysis of DEGs across comparison groups (S2C).

## Slide 3
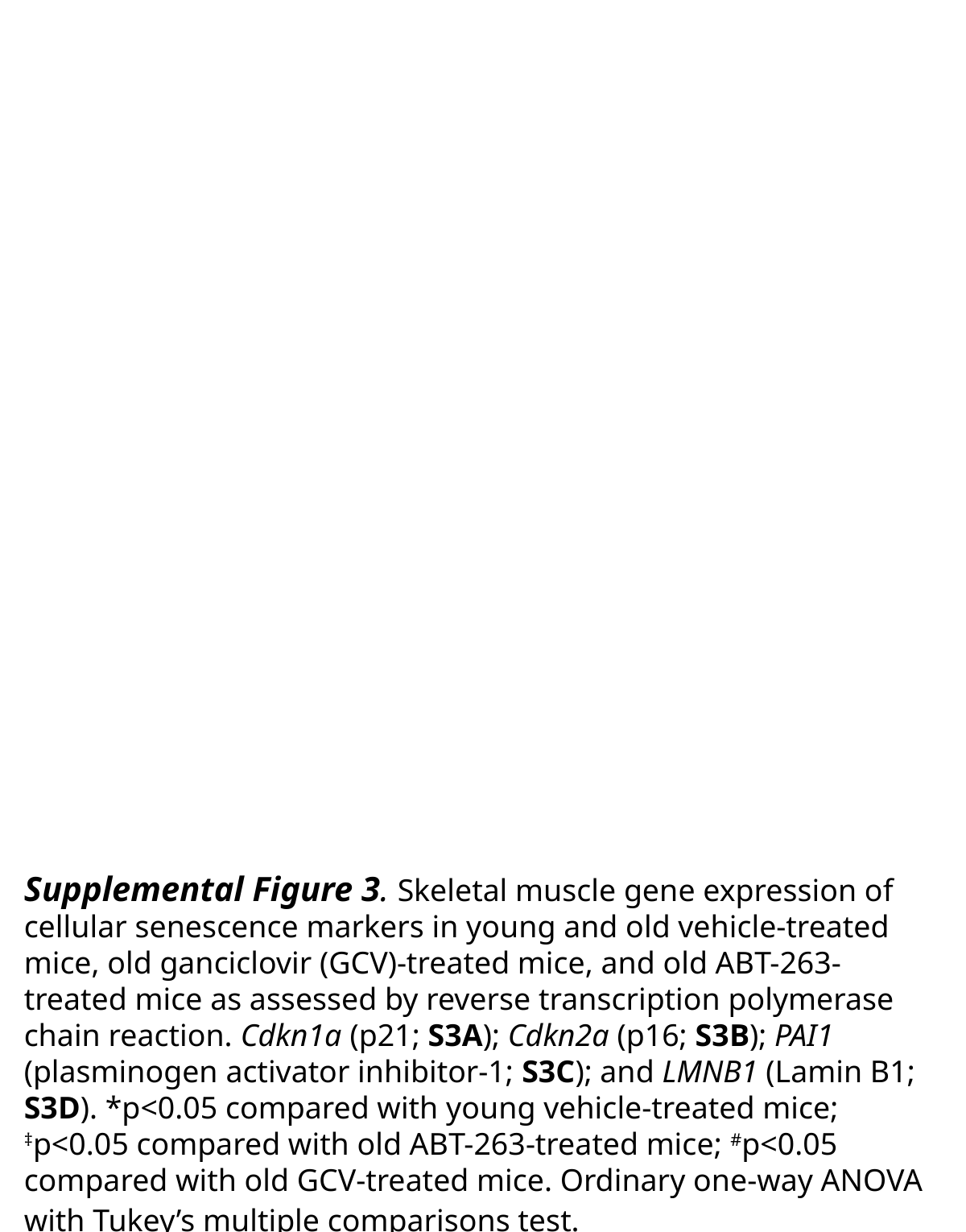

Supplemental Figure 3. Skeletal muscle gene expression of cellular senescence markers in young and old vehicle-treated mice, old ganciclovir (GCV)-treated mice, and old ABT-263-treated mice as assessed by reverse transcription polymerase chain reaction. Cdkn1a (p21; S3A); Cdkn2a (p16; S3B); PAI1 (plasminogen activator inhibitor-1; S3C); and LMNB1 (Lamin B1; S3D). *p<0.05 compared with young vehicle-treated mice; ‡p<0.05 compared with old ABT-263-treated mice; #p<0.05 compared with old GCV-treated mice. Ordinary one-way ANOVA with Tukey’s multiple comparisons test.
