# Supplementary material for: Intermittent Supplementation With Fisetin Improves Physical Function and Decreases Cellular Senescence in Skeletal Muscle With Aging: A Comparison to Genetic Clearance of Senescent Cells and Synthetic Senolytic Approaches
Source: Aging Cell. 2025 May 28;24(8):e70114. doi: 10.1111/acel.70114 (PMC12341784; doi:10.1111/acel.70114)
Supplement: Supplementary file 7 — Data S3. [file ACEL-24-e70114-s001.docx]

Supplemental File 1. **First 3 tabs**: Log 2-fold change and raw p-values for each gene of the 3 comparisons (i.e., old vehicle (Old Veh) vs young vehicle (Young Veh); old fisetin (Old Fis) vs Young Veh; and Old Fis vs Old Veh) identified through bulk RNA sequencing. **4^th^-7^th^ tabs**: Reverse-transcriptase polymerase chain reaction outcomes for Young Vehicle, Old Vehicle, Old GCV, and Old ABT-263 treated mice for genes *Cdkn1a*, *Cdkn2a*, *Pai1*, and *Lmnb1*; each gene is then normalized to GAPDH with corresponding fold chain observed

Supplemental File 2. **First 3 tabs**: Deconvolution analysis Log 2-fold change and raw p-values for each gene determinant by cell type for each of the 3 comparisons (i.e., old vehicle (Old Veh) vs young vehicle (Young Veh); old fisetin (Old Fis) vs Young Veh; and Old Fis vs Old Veh). **4th and 5th tabs**: deconvolution analysis outcomes for genes *Cdkn1a* and *Ddit4*arranged by cell type and comparison. **6^th^ tab:** raw cell proportions and corresponding graphical illustration and statistical analysis (Kruskal-Wallis Test) for each cell type identified through the deconvolution analysis.
